# Mg-Hydroxyapatite Nanorods for Dual Intracellular Doxorubicin Delivery and Osteogenic-Associated BM-MSC Responses

**DOI:** 10.1021/acsabm.5c02324

**Published:** 2026-02-06

**Authors:** Federico Pupilli, Giada Bassi, Marta Tavoni, Monica Montesi, Anna Tampieri, Simone Sprio

**Affiliations:** † Institute of Science, 9327 Technology and Sustainability for Ceramics − National Research Council of Italy (ISSMC−CNR), 48018 Faenza, Italy

**Keywords:** nanorod, hydroxyapatite, Mg^2+^ doping, doxorubicin, drug delivery

## Abstract

Intracellular drug therapies are based on the use of nanocarriers that can successfully penetrate cell barriers and release therapeutic payloads directly inside the cell environment. In this context, hydroxyapatite (HA) nanoparticles provide a particularly promising platform owing to their inherent biocompatibility, bioactivity, and drug-binding capability. This work hence examines anisotropic HA nanorods (NRs), synthesized using hydrothermal methods, with a particular focus on Mg-to-Ca ion substitution, aiming to increase the bioactivity and improve the interaction with therapeutics, specifically targeting intracellular sustained release. Our findings indicate that increasing the extent of Mg doping in apatite NRs induces enhanced cell compatibility and interaction with primary human bone marrow-derived mesenchymal stem cells. Moreover, the doping with Mg^2+^ enhances the NRs capacity to link and release doxorubicin, a widely used antitumor drug, in human osteosarcoma cells. The enhanced functionality is attributed to the Mg^2+^-induced structural disorder at the NR surface, which reduces the crystallinity and increases the number of reactive surface sites. As a result, Mg^2+^ doping has emerged as a promising strategy for optimizing the functional performance of apatite-based nanocarriers, highlighting their potential applications in nanomedicine and precision medicine.

## Graphical abstract

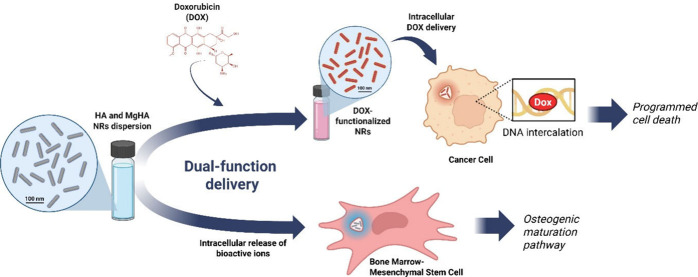

## 1 Introduction

Nanoparticle-based drug delivery systems (DDS) have been intensively investigated, providing substantial enhancements over conventional drug delivery approaches. These systems are designed for precision and personalized therapies, particularly to contrast tumors, inflammatory disorders and cardiovascular diseases.^1–4^ The study of new DDS is particularly focused on the modulation of the size, chemical composition, and morphology of nanocarriers; while size is known to affect the nanoparticle biodistribution,^5,6^ the chemical composition and the surface properties are known to mainly influence nanoparticles cytocompatibility and consequently the cell behavior and fate.^7–9^ In this regard, the use of nanoparticles made of hydroxyapatite (HA) has attracted significant interest in biomedical research owing to their biocompatibility, ability to integrate with biological tissues, and pH-responsive degradability, this latter favoring the prolonged release of bioactive ions such as calcium and phosphate, recognized to be fundamental to cellular metabolism.^2,10,11^ Recently also morphological factors have emerged as relevant to modulate HA nanoparticles’ performance: the HA rod-like structural anisotropy (nanorods: NRs) elicited significant attention in the biomedical field due to enhanced affinity and interaction with biological systems.^12^ In this view, previous works highlighted that HA NRs internalization, as well as the ability to regulate signaling pathways and immune-modulating properties, are affected by diameter and aspect ratio.^13,14^ The interest over HA NRs as DDS resides in their strong chemical affinity with various therapeutic agents such as nucleic acids, proteins, and drugs, specifically influenced by surface functional groups, surface area, pH, and the composition of the surrounding medium.^15^ However, a general drawback related to the use of all nanoparticles is their tendency to aggregate, which hampers their internalization and potential of in vivo driving.^16,17^ Among the various synthesis techniques, hydrothermal synthesis has proven particularly effective for producing HA NPs with high crystallinity, controlled morphology, and tailored chemical composition, all of which are essential for biomedical applications. Moreover, ion doping can be used as a strategy to further modulate physicochemical properties of apatites, particularly the surface composition and structure, both relevant to influence nanoparticles colloidal properties.^18^ Particularly, the doping with Mg^2+^ ions is known to yield defect sites in the apatite lattice, hence leading to enhanced surface hydration and amorphization.^19^ Such effects can markedly affect nanoparticles’ stability and surface charge and ultimately influence the adsorption of relevant biomolecules.^20^ On the other hand, Mg^2+^ doping in apatite-based biomaterials has been widely studied in bone tissue regeneration as its presence is known to promote the nucleation of bone mineral nuclei and to enhance the activation of key osteogenic differentiation pathways that promote osteogenesis and bone tissue regeneration.^21,22^

In the present work, anisotropic HA and Mg-doped HA NRs were synthesized and functionalized with doxorubicin (DOX). Internalization assays were conducted with the human osteosarcoma cell line (MG63) to evaluate their efficacy as an innovative DDS for cell-based anticancer therapies. Preliminary tests carried out with primary human bone marrow-derived mesenchymal stem cells (BM-MSCs) were performed to examine the effects of Mg^2+^ doping on NR uptake and biodegradation as well as its capacity to enhance osteogenic differentiation. While HA NRs have been increasingly studied, Mg-doped HA NRs have not yet been systematically investigated in the context of drug delivery. In this framework, this study introduces a novel approach by exploring their potential as nanocarriers.

## 2 Materials and Methods

### Development of HA and MgHA NRs

Mg-doped HA NRs were synthesized by a hydrothermal method, following similar procedures as previously reported.^19^ Briefly, a phosphate aqueous solution was added slowly to a calcium citrate aqueous dispersion under vigorous magnetic stirring by using a peristaltic pump with a constant flow of 1 mL/min; after phosphate dripping, the pH was maintained at around 12 with the addition of a 1 M solution of sodium hydroxide. To achieve Mg^2+^ ion doping in the apatite structure, magnesium citrate was also added, thus establishing different initial Mg contents, indicated as X_Mg_, [i.e., the molar ratio Mg/(Ca + Mg)], particularly formulations with X_Mg_ = 0.05 and X_Mg_ = 0.10 (hereinafter referred to as MgHA5 and MgHA10, respectively). Mg-free hydroxyapatite NRs (HA) was prepared with the same synthesis route as a control. In all syntheses, the (Ca+Mg)/P ratio was set at 1.67 as a reference value equal to that of stoichiometric HA, and the (Ca+Mg)/citrate ratio was set to 0.8. After addition, the mixture was transferred to a Teflon-lined stainless-steel hydrothermal reactor. The mixture in the reactor underwent hydrothermal treatment at 180 °C for 6 h. Then, the reactor was allowed to spontaneously cool overnight, and the obtained product was purified by using a two-cycle centrifugation-washing process with ultrapure water. Part of the resulting product was redispersed in ultrapure water to form an aqueous suspension for biological evaluation and further functionalization, while the residue was freeze-dried, manually milled, sieved below 50 μm, and finally stored in a dry environment for further physicochemical characterization.

### Physicochemical Characterization

The phase composition of the as-obtained materials was checked by X-ray diffraction (XRD) with a D8 ADVANCE (Bruker, Karlsruhe, Germany) diffractometer using Cu Kα radiation (λ = 1.54178 Å) operated at 40 kV and 40 mA. XRD patterns were acquired in the 10–80° 2θ range at a step size of 0.02° and a scanning time of 1 s. Cell parameters and crystallite size (reported in Table S1) were assessed by full-profile Rietveld analysis of the XRD spectrum (TOPAS v. 4.2, Bruker AXS, Karlsruhe, Germany). Fourier transform infrared (FTIR) spectra in transmission mode (reported in Figure S1) were obtained with a Nicolet 5700 spectrometer, using the KBr pellet method, in the mid-IR range 400–4000 cm^–1^ (64 scans, resolution 4 cm^–1^). Splitting factor indexes (SFI) were calculated from FTIR spectra by applying eq 1, derived by dividing the sum of the absorbance of the peaks at 562 and 602 cm^–1^ from ν_4_(PO_4_) bond bending by the absorbance of the minimum between these two peaks, following similar procedures from the literature.^23,24^

1 $$\mathrm{Splitting Factor Index}=\frac{\left(A_{\max\left(602\right)}+A_{\max\left(567\right)}\right)}{A_{\min\left(590\right)}}$$

The chemical analysis was performed on dried samples (∼10 mg) using inductively coupled plasma optical emission spectroscopy (ICP–OES, Agilent 5100, Santa Clara, CA, Varian, Palo Alto, CA, USA) and primary standards (1000 ppm, Fluka). For this analysis, the samples were dissolved into 1 mL of nitric acid and then diluted in 50 mL of ultrapure water.

### Morphological Characterization

The materials morphology was evaluated with scanning electron microscopy with a field-emission microscope (FEG-SEM, mod. ΣIGMA, ZEISS NTS Gmbh, Oberkochen, Germany), operating at a 4 kV acceleration voltage, with a working distance of 3.5 mm, and acquired using the InLens detector at 150K× magnification. HA and MgHA NRs were dispersed in ultrapure water to a concentration of 0.1 mg/mL. Afterward, 2 μL of the NRs suspension was deposited on a microscope slide coverslip mounted on an aluminum stub and dried at room temperature. Once the samples were dried, they were sputter-coated (Polaron E5100, Polaron Equipment, Watford, Hertfordshire, UK) with 2 nm of a Pt/Pd (80:20) alloy to provide electrical conductance. To obtain NRs dimensional analysis, ImageJ was used to analyze grayscale SEM images in the TIFF format using the following sequential steps: Set the Scale, Brightness/Contrast, and Threshold. The width and length of NRs were measured, and then, the aspect ratio was calculated as the ratio between the length and width of each NR. Here, 100 NRs were measured to provide data with suitable statistical significance. Subsequently, length and width data were furtherly elaborated using the ORIGIN 8.1 software (results reported in Figure S2). Gaussian fitting of the size distribution was used to determine the mean width, length, and aspect ratio. NRs size and agglomeration behaviors were obtained by dynamic light scattering of redispersed samples in ultrapure water, measured with a Zetasizer Nano ZS instrument (Malvern Ltd., Worcestershire, U.K.). For the analysis, samples were dispersed in ultrapure water at a concentration of 0.2 mg/mL at pH 7.2. ζ-Potentials were quantified by laser Doppler velocimetry as electrophoretic mobility at 25 °C using a disposable electrophoretic cell (DTS1061, Malvern Ltd., Worcestershire, UK) of three separate measurements (40 runs each).

### Degradation Test

The degradation rate was evaluated by mixing 30 mg of lyophilized NRs in 9 mL of 10 mM HEPES buffer (pH 7.4) or 10 mM acetate buffer (pH 5.0). The release of ions from the samples was evaluated by placing 10 mg of the obtained lyophilized powders in a Transwell with a 0.4 μm pore membrane insert, immersing in 9 mL of 10 mM HEPES buffer (pH 7.4) or 10 mM acetate buffer (pH 5.0) kept at 37 °C and gently shaking at 200 rpm. At scheduled times (i.e., after 0.25, 1, 2, 3, 7, and 10 days), the solution was removed and filtered with a 0.20 μm pore size syringe filter, and 3 mL of fresh solution was added. The supernatants containing the released ions (prior dissolution in 1 wt % ultrapure nitric acid) after the prefixed times were analyzed by ICP–OES, in triplicate, for the quantitative determination of Ca, Mg, and P. Releases in wt % are reported in Figure S3.

### Cell Culture

Bone marrow-derived mesenchymal stem cells (BM-MSCs), purchased from American Type Culture Collection (ATCC) (PCS-500-012), were cultured in Alpha-MEM GlutaMAX (Gibco, Carlsbad, CA) supplemented with 15% fetal bovine serum (FBS), 1% penicillin-streptomycin (Pen/Strep, 100 U/mL-100 μg/mL), and 10 ng/mL of human basic fibroblast Growth Factor (hbFGF). Human Osteosarcoma MG63 cell line was purchased from ATCC (CRL-1427) and cultured in DMEM-F12/GlutaMAX (Gibco Carlsbad, CA), supplemented with 10% FBS and 1% of Pen/Strep. Both cells were grown in an incubator at 37 °C, 5% CO_2_, and controlled humidity conditions. Cells were detached from culture flasks by trypsinization, centrifuged, and resuspended in fresh media. Cell number and viability were assessed with a trypan-blue dye exclusion test.

### NRs Handling Procedure for Cell Culturing

The cytocompatibility and gene expression of the obtained NRs were performed on BM-MSCs seeded at a density of ∼2500 cells/cm^2^. The day after, different concentrations of HA and MgHA NRs (200, 100, 50, 20 μg/mL) were added to the cell culture by dilution in the media. NRs suspension in the medium was vortexed before being added to the cells. The osteogenic medium composed of Alpha-MEM GlutaMAX (Gibco, Carlsbad, CA) supplemented with 15% FBS, 1% Pen/Strep, 10 mM β-glycerophosphate, 50 μg/mL ascorbic acid, and 100 nM dexamethasone was used in this study to test the potential osteogenic induction by NRs. The cells were incubated at 37 °C, 5% CO_2_, and controlled humidity conditions for up to 7 days. Cells grown without NRs addition (cells only) were used as a control. The cells were analyzed in terms of cell viability and proliferation, cell morphology, and gene expression.

### FITC-Conjugation of HA and MgHA NRs

Following a similar procedure in the literature,^25^ 1.75 mg of fluorescein isothiocyanate (FITC, Sigma-Aldrich, ≥90%) and 10.84 mg of (3-aminopropyl)-triethoxysilane (APTS, Sigma-Aldrich, ≥99%) were weighed and dissolved in 2 mL of anhydrous ethanol by magnetic agitation in the dark at RT for 24 h. Colloidal dispersions of HA and MgHA NRs in 300 mM citrate were prepared. Here, 0.625 mL of the prepared dispersion was centrifuged at 14,000 rpm for 15 min. The supernatant was removed, and to the pellet were added 25 mL of ethanol together with 10 μL of *n*-butylamine (Sigma-Aldrich, ≥99.5%) to improve NRs dispersion in ethanol. The content was transferred to a glass bottle, to which 0.5 mL of the FITC-APTES conjugate was added, together with 0.5 mL of ammonia and 0.5 mL of tetraethyl-orthosilicate (TEOS, Sigma-Aldrich, ≥99%). The solution was mixed by magnetic agitation for 24 h at RT in the dark. Subsequently, the content was centrifuged and washed twice with ethanol aliquots and later resuspended in 0.5 mL of ultrapure water to obtain a final concentration of 25 mg/mL.

### Cell Viability Analysis

The cell viability and proliferation were measured by MTT assay and live/dead assay.^26,27^ Briefly, the MTT reagent (3-(4,5-dimethylthiazol-2-yl)-2,5 diphenyltetrazolium bromide) was prepared at 5 mg/mL in 1× phosphate buffer saline (PBS). Cells seeded in 96-well plates were incubated with the MTT reagent 1:10 for 2 h at 37 °C. The medium was removed, and the resulting formazan crystals were dissolved with 1 mL of dimethyl sulfoxide for 15 min under stirring conditions. Indeed, the alive cells are able to metabolize the MTT reagent producing formazan crystals that are dissolved in DMSO obtaining a pinkish solution which can be read at λ_max_ of 570 nm, using a Multiskan FC microplate photometer (Thermo Scientific). Absorbance correlates directly with the quantity of metabolically active cells. Cell viability was reported as % with respect to cells only. The samples were analyzed in quadruplicate at days 1, 3, and 7 (N = 4). The qualitative determination of cell viability was assessed by the live/dead assay based on the simultaneous detection of live and dead cells by green-fluorescent calcein-acetoxymethyl (AM) and red-fluorescent ethidium homodimer-1, respectively. Briefly, at day 1 of culture, cells were washed in PBS 1x for 5 min and stained with Calcein-AM 2 μM and ethidium homodimer-1 4 μM for 15 min at 37 °C. Subsequently, cells were again washed in PBS 1x for 5 min and viewed under inverted Ti-E fluorescence microscopy (Nikon, Japan). Two samples per each group were analyzed (N = 2).

### Actin/DAPI Staining

At 3 days of culture in the presence of NRs, the cells were fixed, permeabilized, and stained by Actin and DAPI staining. Briefly, a wash with PBS 1X was done, followed by incubation for 10 min with 4% w/v paraformaldehyde (PFA) (Sigma-Aldrich) at room temperature (RT). An additional wash with PBS 1X was done before the addition of 0.1% (v/v) Triton-X (Sigma-Aldrich) for 5 min at room temperature. Another wash with PBS 1X was performed, and the cells were stained with ActinRed555 Ready Probes reagent (Invitrogen) to stain the cytoskeleton, following the manufacturer’s instructions. The counterstaining of cell nuclei was performed using 4′,6-diamidino-2-phenylindole (DAPI) (600 nM) (Invitrogen) following the manufacturer’s instructions.^28,29^ Images were acquired using an Inverted Ti-E fluorescent microscope. One biological replicate for each sample was performed (N = 1).

### Cell Internalization of FITC-Labeled NRs

NRs internalization studies were performed on BM-MSCs seeded at the same density as that previously reported. The day after, HA and MgHA NRs FITC labeled following the procedure reported above were added to the cell culture media reaching 200 μg/mL NRs concentration. Cells were incubated for 6 and 24 h under standard culture conditions. Following incubation, cells were washed twice with PBS to remove unbound NRs, fixed, and stained following the same procedure as reported above. For each NRs type and time point, 10 images captured at 10× magnification were randomly selected from the total data set and analyzed in ImageJ. Images were split and converted to 8-bit and channels. DAPI (nuclear marker) and FITC (NRs fluorescence) channels were processed separately. Nuclei were smoothed and thresholded. Objects with an area smaller than 800 pixels^2^ were excluded, and the remaining objects were counted as nuclei. The NRs channel was background-subtracted and thresholded with a fixed intensity window (lower = 74; upper = 255) applied identically to all images to ensure comparability. A cell was considered positive for NRs internalization if FITC fluorescence was detected within the actin-defined cytoplasmic region. For each image, the total number of cells N_tot_ and the number of nanoparticle-positive cells N_pos_ were recorded, and the positivity ratio (N_pos_/N_tot_) was calculated. The percentage of positive cells was averaged across the 10 randomly selected images and reported as mean ± SD for each NRs type and incubation time. Two biological replicates for each sample were performed (N = 2).

### Quantitative Real-Time Polymerase Chain Reaction (qPCR)

At days 7 and 14, cells grown in the presence of the different concentrations of HA and MgHA NRs were analyzed, using cells only as the control group. The lysis of the samples was performed by using the Tri Reagent (Invitrogen) followed by the Total RNA extraction by using the Direct-zol RNA Mini Prep kit (Zymo Research), according to the manufacturer’s instructions. Then, RNA quantification and purity degree were evaluated with a NanoDrop one microvolume UV–vis spectrophotometer (Thermo Scientific). A high-capacity cDNA reverse transcription kit was used to obtain a double-stranded cDNA starting from 500 ng of purified RNA, following the manufacturer’s instructions. The gene expression was evaluated using TaqMan gene expression assays (Applied Biosystems), for runt-related transcription factor 2 (RUNX2, HS00231692_M1), alkaline phosphatase (ALP, HS01029144_M1), bone morphogenetic protein 2 (BMP-2, HS00154192_M1), osteocalcin (BGLAP, HS01587814_G1), osteopontin (SPP1, HS00959010_M1) and glyceraldehyde 3-phosphate dehydrogenase (GAPDH, HS99999905_M1), used as a housekeeping gene. A biological duplicate was performed for each sample (N = 2), and each replicate was analyzed in three technical replicates. Experiments were performed by using the QuantStudio 1 Real-Time PCR System (Applied Biosystems), and the relative quantification of the target genes was assessed using the comparative threshold (Ct)­method (ΔΔCt), where relative gene expression level equals 2^–ΔΔCt^.^25,30^

### Development of DOX-Functionalized Apatite NRs

Initially, adsorption kinetics were determined to identify the best procedure for DOX adsorption (Figure S4). In this context, 20 mg/mL of HA colloidal dispersion in a 134 mM sodium citrate solution was combined with a 400 ppm solution of DOX in a 1:1 ratio. The mixture was then placed in a horizontal incubator at 37 °C in the absence of light, as DOX is susceptible to photolytic degradation.^31^ At predefined time points (30 min, 1 h, 2 h, 3 h, 4 h, and 6 h), the adsorption was stopped by centrifugation of the dispersion at 12,000 rpm for 5 min. The supernatant was collected, and the DOX content was measured by UV–vis spectroscopy with a Cary 1E UV–vis spectrophotometer (Varian, USA) at a wavelength of λ = 481 nm. The DOX content was determined by using a calibration curve that had been previously obtained. The discrepancy between the initial quantity of DOX and the subsequent value was utilized to determine the amount of DOX that was adsorbed. The equilibrium concentration was then estimated by dissolving the dispersion in an acidic solution in order to determine the overall content of DOX. From adsorption kinetics and isotherms, the following optimized procedure was employed to develop DOX-functionalized NRs.

To a 10 mL 400 ppm DOX solution, 10 mL of a 20 mg/mL NRs colloidal dispersion in 134 mM sodium citrate was added drop by drop. The mixture was then placed in a horizontal incubator at 37 °C for 2 h. Afterward, the functionalized NRs underwent centrifugation at 12,000 rpm for 5 min. The supernatant was discarded, and the NRs were subsequently rinsed with ultrapure water using the same centrifugation conditions. Subsequently, the pellet was resuspended in ultrapure water and passed through 0.45 μm pore size syringe filters for further characterization and biological assessment. Part of the resuspended dispersion was lyophilized to determine the DOX loading release profile.

### Determination of DOX Loading and Release on Obtained NRs

To determine the DOX loading percentage, around 20 mg of lyophilized DOX-functionalized NRs was dissolved in HCl 0.1 M solution, and the DOX loading quantity was evaluated with UV–vis analysis following eq 2.

2 $$\mathrm{DOX loading (wt \%)}=\frac{\mathrm{mass DOX (mg)}}{\mathrm{mass DOX (mg)}+\mathrm{mass NPs (mg)}}\times 100$$

For the DOX release experiment, 20 mg of lyophilized DOX-functionalized NRs was resuspended either in 5 mL of HEPES buffer (10 mM, pH 7.4) or 5 mL of acetate buffer (10 mM, pH 5.0), reaching a NRs concentration of 4 mg/mL. Suspensions were constantly stirred at 37 °C. At scheduled times, the samples were centrifuged, and 1 mL of supernatants was collected and replaced with fresh buffer. The supernatant was analyzed by UV–vis spectroscopy. DOX released (wt %) was described as the ratio between the amount of drug released at time t (Q_(t)_) and the drug loading capacity, Q_max_, based on eq 3:

3 $$\mathrm{DOX released (wt \%)}=\frac{Q_{\left(t\right)}}{Q_{\max}}\times 100$$

To assess the drug release kinetics, experimental data were analyzed through nonlinear least-squares regression utilizing OriginPro 8.1 (OriginLab Corp., Northampton, MA, USA). Model parameters were fitted through the minimization of the residual sum of squares (RSS), while the goodness of fit was evaluated using the correlation coefficient R^2^ (COD) values. Among the models tested, the Korsmeyer–Peppas (KP) model showed satisfactory fit to the DOX release data. The KP model is described by eq 4:

4 $$\frac{M_{\left(t\right)}}{M_{\infty }}=Kt^{n}$$

where M_t_/M_∞_ represent the released fraction of payload at time t, while K and n are the rate constant and release exponent of the Korsmeyer–Peppas model, respectively.

### Biological Analysis of DOX-Functionalized NRs

The potential activity of DOX-functionalized NRs as drug delivery system was investigated on osteosarcoma MG63 cells plated at a density of ∼5000 cells/cm^2^ and on BM-MSCs as a healthy control at the same cellular density previously reported. The seeding procedure was carried out in the same manner as previously described. The potential action of DOX-functionalized NRs was investigated in terms of cell viability on MG63 and BM-MSCs by MTT assay and cellular uptake by image acquisition of red fluorescent drug internalization in cell nuclei. Briefly, the MTT assay was performed as reported in the above-mentioned paragraph, following the manufacturer’s instruction, testing the cytotoxicity of DOX-functionalized HA and MgHA NRs at a fixed DOX concentration of 10 μg/mL, compared to free DOX at the same concentration, unloaded HA and MgHA NRs at the same equivalent NRs concentration (corresponding to 323 and 207 μg/mL of HA and MgHA NRs, respectively) and cells only, after 1, 2, and 3 days of culture.

The cellular uptake of DOX-functionalized HA and MgHA NRs was investigated on MG63 cells by image acquisition of red fluorescent drug internalization in the cell after 6 h of cell culture, compared to free DOX and cells only. The cells were plated on collagen Type I-coated glass slides in 24 well plates, and the day after, the DOX-functionalized HA and MgHADOX were added at 10 μg/mL concentration. After 6 h, the media were removed, and the cells were washed twice in PBS 1X and fixed in PFA 4% for 15 min. The counterstaining of cell nuclei and the cytoskeleton was performed by DAPI/ActinGreen staining (ActinGreen488 Ready Probes reagent, Invitrogen). Two biological replicates for each sample were performed (N = 2).

The cytotoxicity of DOX-functionalized HA and MgHA NRs was tested on a wide concentration range of DOX (0.138, 0.416, 1.25, 3.75, 7.5, 15, 30, and 60 μg/mL) on MG63 cells at day 1 of culture, compared to free DOX and to NRs at the highest equivalent concentration ([DOX]: 60 μg/mL, corresponding to 2 and 1.3 mg/mL of HA and MgHA NRs, respectively) by MTT assay, following the manufacturer’s instructions, as reported above. Three biological replicates for each sample were performed (N = 3).

### Statistical Analysis

Results of the MTT assay and cell positivity to NRs were expressed as mean value ± standard deviation (SD), as plotted on the graphs. Results of gene expression were graphically reported as fold-change expression relative to the cells only ± SD. Statistical analysis was performed by two-way ANOVA, followed by Bonferroni’s posthoc test using GraphPad Prism software (version 8.0), with statistical significance set at p ≤ 0.05.

## 3 Results and Discussion

### 3.1 Physicochemical Characterization

The XRD patterns of all of the obtained materials are reported in Figure 1, showing that apatite is the only crystalline component. In all doped materials, Mg^2+^ doping induces a general broadening of the XRD peaks, which could be correlated to the Mg-to-Ca substitution in the apatitic lattice. The likely substitution of smaller Mg^2+^ ions induced greater distortion of the crystalline domains and a reduction in overall crystallinity, as evidenced by the splitting factor index reduction from FTIR data (Table S1, Figure S1) and by crystallite size analysis along the ab plane and c-axis through peak profile fitting. While HA NRs displayed greater crystallite length along the c-axis (D002) (Table S1), Mg^2+^ doping progressively inhibited this preferential growth, promoting the formation of more isotropic apatitic crystals. The results of the chemical analysis conducted via ICP-OES (reported in Table 1) further verify the substitution of Ca^2+^ with Mg^2+^. Such a hypothesis is evidenced by the actual molar ratio X_Mg_, which increases with the nominal Mg^2+^ doping, although it exhibits slightly lower values than those nominally introduced. Moreover, the Ca/P ratio decreases progressively with increasing Mg^2+^ content, far lower than the stoichiometric HA Ca/P ratio, which is 1.67.^19^ Since all samples retain a pure apatitic phase, these low ratios suggest a composition far from stoichiometry, consistent with Mg^2+^ incorporation into the apatite lattice. The stable (Ca+Mg)/P ratio, combined with the increasing Mg wt %, further supports the possibility of Mg^2+^ substituting for Ca^2+^ in the structure.

**Figure 1:**
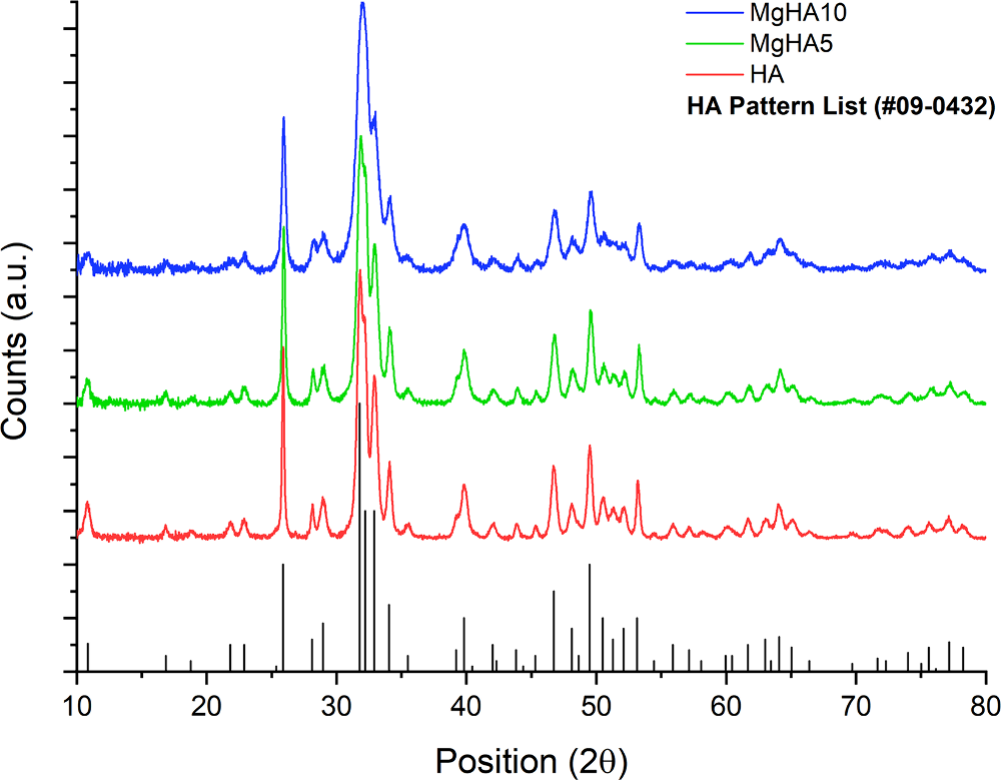
XRD patterns of the obtained apatite NRs (stoichiometric HA pattern shown in black corresponds to the PDF file #09-0432).

**Table 1:** Chemical Analysis of Obtained HA and MgHA NRs

Sample	nominal X_Mg_%	actual X_Mg_% (ICP)	Ca/P (mol) (ICP)	(Ca + Mg)/P (mol)
HA	0	–	1.62 ± 0.01	1.62 ± 0.01
MgHA5	5	3.48 ± 0.01	1.56 ± 0.01	1.62 ± 0.01
MgHA10	10	6.49 ± 0.01	1.48 ± 0.01	1.59 ± 0.01

To better understand how Mg^2+^ doping affects the crystal growth, observation of NRs morphology was inspected in the SEM micrographs (Figure 2). In the specific case of hydrothermal synthesis, the substitution of Ca^2+^ with Mg^2+^ ions inhibited anisotropic crystal growth, as seen by looking at the decreasing aspect ratio values (Table 2), resulting from a pronounced reduction in the NR length and a comparatively minor decrease in the width. Although a minor tendency for aggregation is visible for Mg^2+^-doped HA NRs, DLS data in Table 2 show the formation of homogeneous and monodisperse NRs in all cases, as also confirmed by the low polydispersity index (PDI). To further study the effect of Mg^2+^ doping, ζ-potential analyses were performed in ultrapure water (Table 2), showing negative values that slightly increased with Mg^2+^ content. These results are consistent with the differences observed among HA, MgHA5, and MgHA10 NRs in terms of hydrodynamic diameter and PDI. Such variations can be ultimately related to the differences in terms of surface/volume ratio (derived by the smaller size of MgHA NRs, as seen in Table 2) together with differences in ζ-potential surface potential (Table 2).

**Figure 2:**
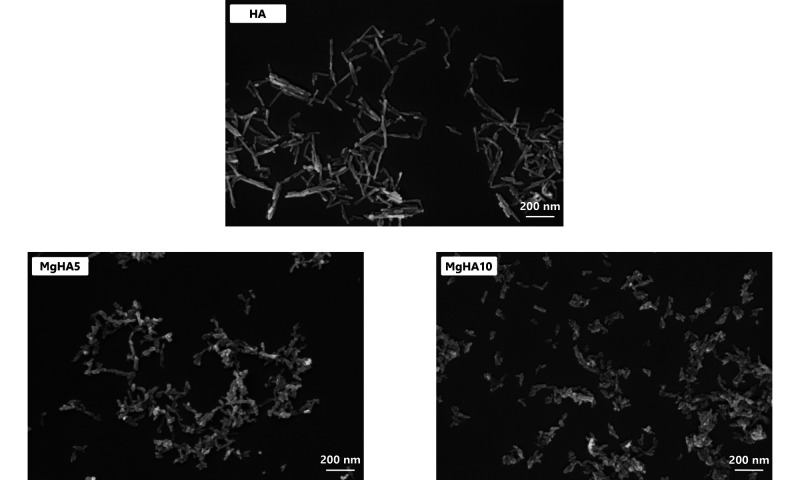
SEM micrographs of the NRs synthesized (scale bar: 200 nm).

**Table 2:** NRs Dimensional Parameters Calculated from DLS and SEM Analysis

Sample	Hydrodynamic diameter (nm)	PDI	ζ-potential (mV)	Avg. Length (SEM) (nm)	Avg. Width (SEM) (nm)	Avg. Aspect ratio (SEM)
HA	86.3 ± 0.6	0.13 ± 0.08	–23.4 ± 0.6	107 ± 2	27 ± 2	4.53 ± 0.07
MgHA5	93.4 ± 0.3	0.11 ± 0.02	–22.1 ± 0.1	74 ± 1	26.4 ± 0.5	2.71 ± 0.05
MgHA10	114 ± 1	0.27 ± 0.03	–19.4 ± 0.1	54 ± 1	21.2 ± 0.3	2.49 ± 0.09

In view of conducting biologic tests with cells, analysis of ions release from HA NRs was carried out under physiological pH and in an acidic environment comparable to that found in lysosomes, where NRs dissolve following cellular uptake (reported in ug/mL in Figure 3 and in wt % in Figure S3).^32,33^ To target their application as DDS, the pH-dependent degradability plays a critical role to ensure the timely release of therapeutic agents at the target site.^31^ In this context, the amount of phosphate ions released (μg/mL) directly reflects the degradation rate of apatitic-based NRs. While overall degradation is relatively low, mainly as a result of NR aggregation in the media, it increases with a higher Mg^2+^ content, likely reflecting the associated reduction in NRs crystallinity. Furthermore, while calcium release appears comparable among HA, MgHA5, and MgHA10, notable variations emerge when magnesium release from the doped NRs is taken into account. As previously reported for anisotropic Mg-doped HA NRs,^19^ magnesium is predominantly located at the NR surface, leading to rapid release in acidic media. Such behavior is highly advantageous for multifunctional nanoplatforms, as magnesium release promotes osteogenic responses, and the transient surface environment of the NRs can facilitate the rapid detachment and delivery of adsorbed therapeutic agents.

**Figure 3:**
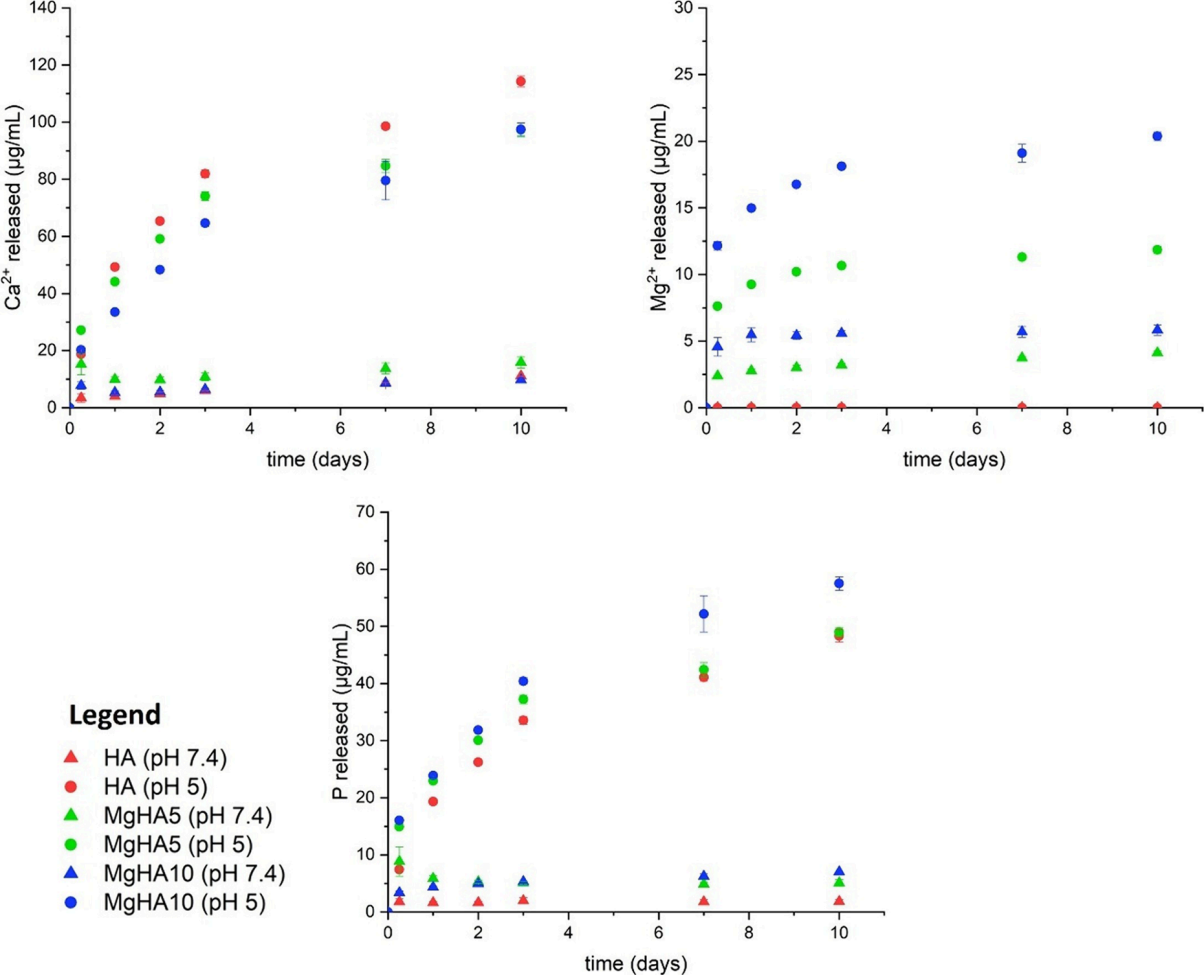
Ionic releases of studied NRs at physiological (HEPES 10 mM buffer pH: 7.4; acetate 10 mM buffer pH: 5).

### 3.2 Effect of Mg Doping on Cell Viability and Morphology

To investigate the biological activity of HA and MgHA NRs, BM-MSCs were cultured in the presence of different NRs concentrations (200 μg/mL; 100 μg/mL; 50 μg/mL; 20 μg/mL);^34^ cells only were used as a control. Cell viability and proliferation was assessed qualitatively at day 1 of cell culture in the presence of NRs with live/dead assay and by quantitatively evaluating metabolically active cells at days 1,3, and 7, with the employment of MTT assay. Cell morphology was evaluated at day 3 by staining with fluorescent dye phalloidin and DAPI. Live/dead assay shows no signs of cytotoxicity at day 1 of NRs exposure for all concentrations tested (Figure 4). This result was further confirmed by quantification of cell viability (Figure 5) and proliferation (Figure S5) assessed by the MTT test. While on day 1 there is no presence of cytotoxicity at all the concentrations tested, starting from day 3 a slight dose-dependent cytotoxicity was observed, especially for HA and MgHA5 NRs.

**Figure 4:**
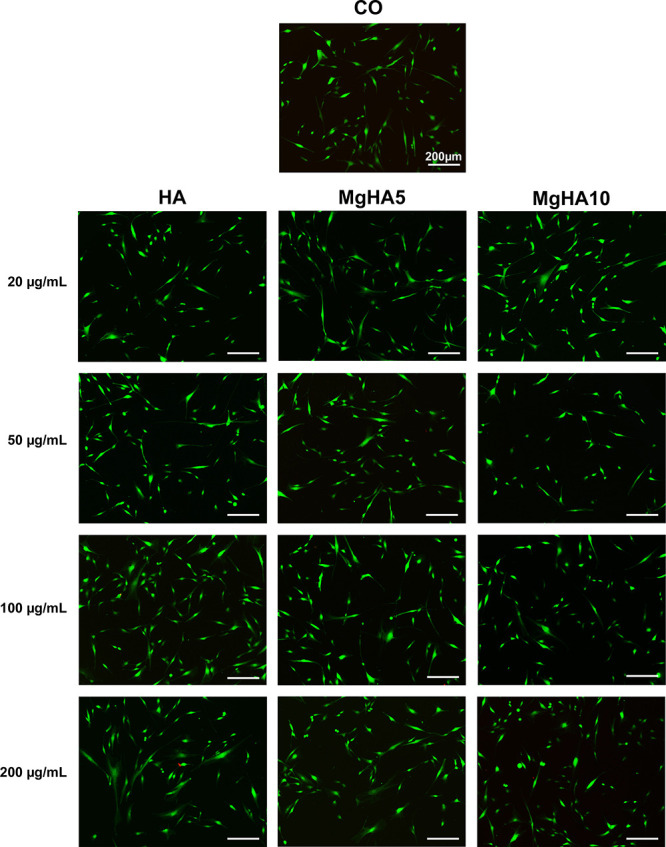
Live/Dead assay on BM-MSCs after 1 day in contact with apatite NRs at different concentrations. Cells Only (CO) were used as control. Live cells in green, dead cells in red. Scale bar: 200 μm.

**Figure 5:**
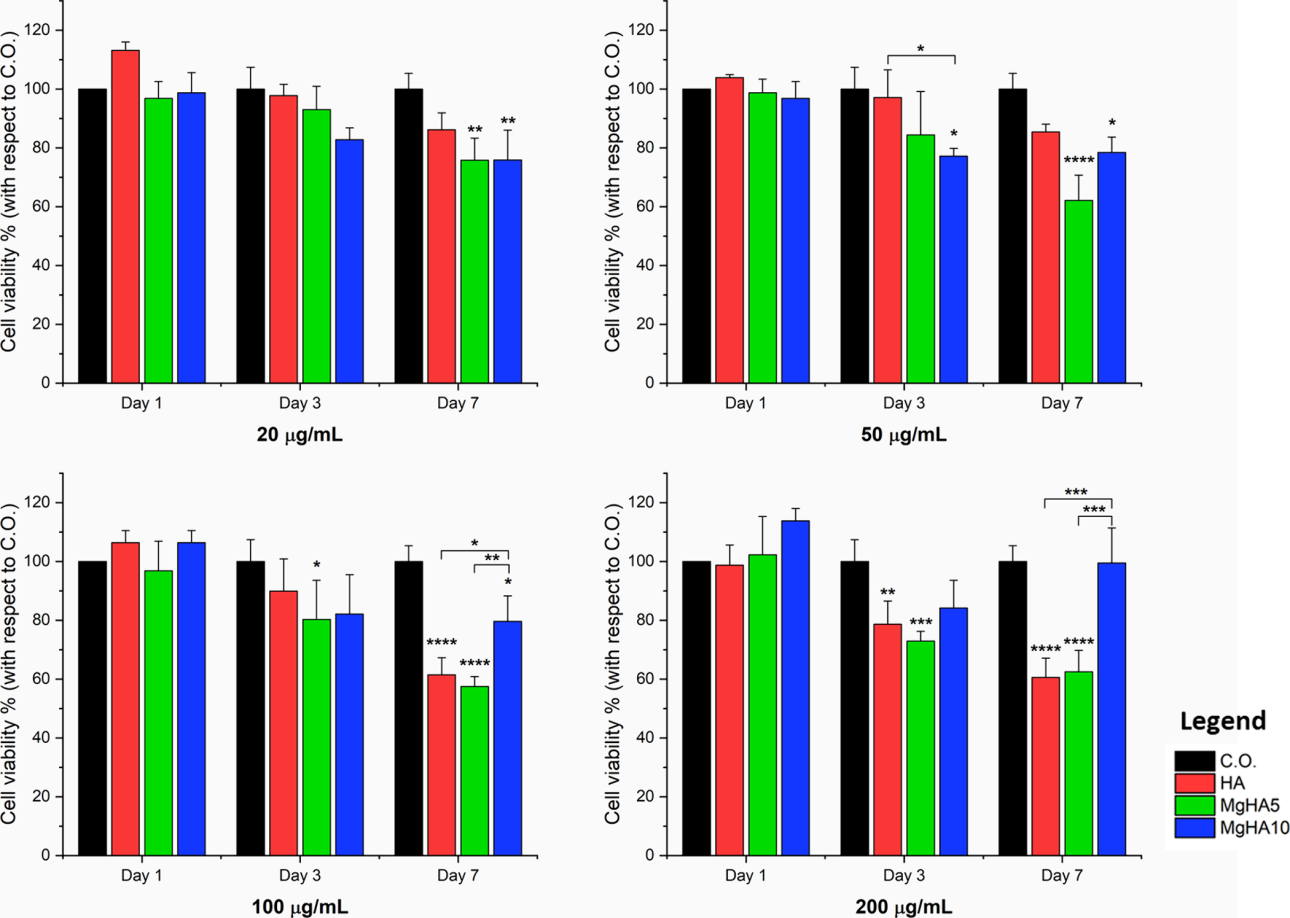
Cell viability analysis. MTT assay was performed after 1 day, 3 days, and 7 days of BM-MSCs culture with 20, 50, 100, and 200 μg/mL of HA, MgHA5, and MgHA10 NRs. Cells Only (CO) were used as control (mean ± standard error; *p value ≤ 0.05; **p value ≤ 0.01; ***p value ≤ 0.001; ****p value ≤ 0.0001). Asterisks placed above the histograms, without connecting lines, indicate statistical significance over the cells only sample.

It is possible to observe a statistically significant decrease in cell viability at day 3 induced by the presence of NRs at 100 and 200 μg/mL, while no statistical difference in terms of cytotoxicity is seen with sample MgHA10, even at higher NRs concentration (MgHA5 100 μg/mL p value ≤ 0.05; HA and MgHA5 200 μg/mL p value ≤ 0.01 and ≤ 0.001, respectively). Although at day 7 100 μg/mL of MgHA10 NRs showed a decrease in cell viability compared to the cells only (*p* ≤ 0.05), this reduction was significantly lower than that induced by the other NRs (p ≤ 0.05 vs HA and p ≤ 0.01 vs MgHA5). Notably, MgHA10 NRs at 200 μg/mL preserved cell viability at levels similar to the untreated cells, and lower concentrations consistently supported high viability. These results are notably different from those of HA and MgHA5, both of which result in a considerable reduction of cell viability compared to cells only at both the concentrations (HA and MgHA5 at 100 and 200 μg/mL feature p value ≤ 0.0001), and such effects can be further observed by looking at the cell proliferation assay (reported in Figure S5). All of the NRs, nevertheless, show a progressive proliferation over time, as further confirmed by a statistically significant increase in cell proliferation, particularly relevant at day 7 with respect to day 1. The intrinsic cytocompatibility of MgHA10 NRs with respect to the other samples could be hence due to the improved degradability in a biological environment caused by higher Mg^2+^ doping, as already confirmed by ion release data shown in Figure 3. It is widely recognized that the role of bioactive ions, such as Ca^2+^, Mg^2+^ and PO_4_^3–^ among others, favor cell proliferation and osteogenesis.^35–38^ In bone tissue regeneration, enhanced degradability is a desirable feature for calcium phosphate-based materials. The incorporation of bioactive ionic dopants, such as Mg^2+^, enables their controlled release once NRs are internalized in bone relevant cells. When released into the intracellular environment, these ions act as cofactors for enzymatic activities, as they are responsible for the structural stability of proteins, and they regulate numerous biochemical reactions in the cell.^19,39^ In the present work, the considerable increase in cell viability, observed with MgHA10 at the highest concentration, may account for this phenomenon. Furthermore, the combined evaluation of cytocompatibility (Figure 5) and Mg^2+^ ion release profiles (Figure 3 and Figure S3) demonstrates that the amount of magnesium released from the highly doped MgHA10 NRs remains well within a cytocompatible range. Even under the most concentrated experimental conditions, the cumulative Mg^2+^ release from MgHA10 NRs—calculated from the highest Mg^2+^ release value (wt %) reported in Figure S3 for samples tested at 200 μg/mL in the MTT assay—did not exceed approximately 3.9 μg/mL (≈0.16 mM). This concentration is significantly lower than the levels associated with cytotoxicity or hypermagnesemia, as previous studies have shown that magnesium concentrations between 20 and 40 mM can markedly reduce mesenchymal stem cell viability, and subtoxic adverse effects on differentiation and calcium signaling can appear from ≈1.3 mM, intensifying by 1.8 mM.^40,41^ This evidence, together with the maintained cell viability and proliferation, indicates that MgHA10 NRs degrade in a controlled manner without causing an ionic imbalance or toxicity to surrounding cells. The degradation behavior and Mg^2+^ release kinetics support the long-term safety and biocompatibility of the material, mitigating concerns about systemic Mg^2+^ overload in potential in vivo applications. More detailed analysis of the molecular pathways that are triggered by the ions in the cell should be carried out to fully confirm our hypothesis.

The arrangement of the cytoskeletal framework, composed of actin filaments, is crucial for preserving and regulating the shape and stability of cells.^42,43^ In this study, the morphology of BM-MSCs grown in contact with HA and MgHA NRs was examined. The cells remained firmly attached to the surface and were not adversely affected by the presence of the NRs, even at higher concentrations, after 3 days (Figure 6). Furthermore, the cells retained their characteristic spindle/fibroblast-like shape without any discernible variation across the groups. Given the healthy nuclear morphology observed, we can confirm the absence of any apoptotic or stress-related phenomena (Figure 6).

**Figure 6:**
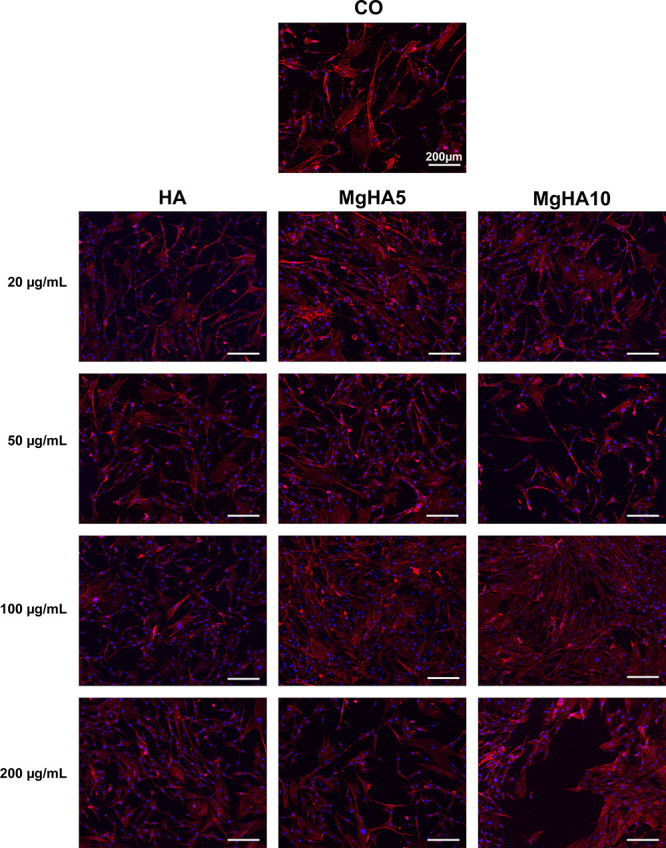
Analysis of cell morphology by Actin and DAPI staining at day 3 of culture. Cells Only (CO) were used as control. F-actin filaments in red; cell nuclei in blue. Scale bars 200 μm.

### 3.3 NRs Internalization in Cells

To further investigate NRs uptake behavior after Mg^2+^ doping, FITC was chemically conjugated to NRs surfaces and incubated with BM-MSCs for 6 h (Figure 7a) and 24 h (Figure 7b) at the concentration of 200 μg/mL, which was chosen based on favorable cell viability results. The uptake of NRs was then consistently monitored using fluorescence microscopy. FITC conjugation is a common and effective approach for assessing NRs cell internalization. However, it can alter key physicochemical properties, such as size and surface charge, thereby affecting cellular interactions and uptake efficiency.^44,45^ Thus, the present results should be considered preliminary, providing a screening of size-dependent internalization behavior modulated by Mg^2+^ doping. In the present work, cells treated with NRs exhibit a uniform mechanism of NR uptake plausibly through endocytosis, probably clathrin and caveolin mediated, based on the dimensions of the NRs.^17,46^ The presence of NRs did not negatively affect the cell cytoskeleton, showing a morphology similar to that of the control group (Figure 7). However, it can be noted that in all samples only a small fraction of NRs was effectively present inside cells. This may be attributed to factors related to the FITC functionalization process, such as possible NR agglomeration, and partial masking of surface functional groups during conjugation. These effects could limit NR availability for uptake, although they do not diminish the value of FITC functionalization as a practical screening tool. Rather, they highlight the need for careful interpretation of the results.

**Figure 7:**
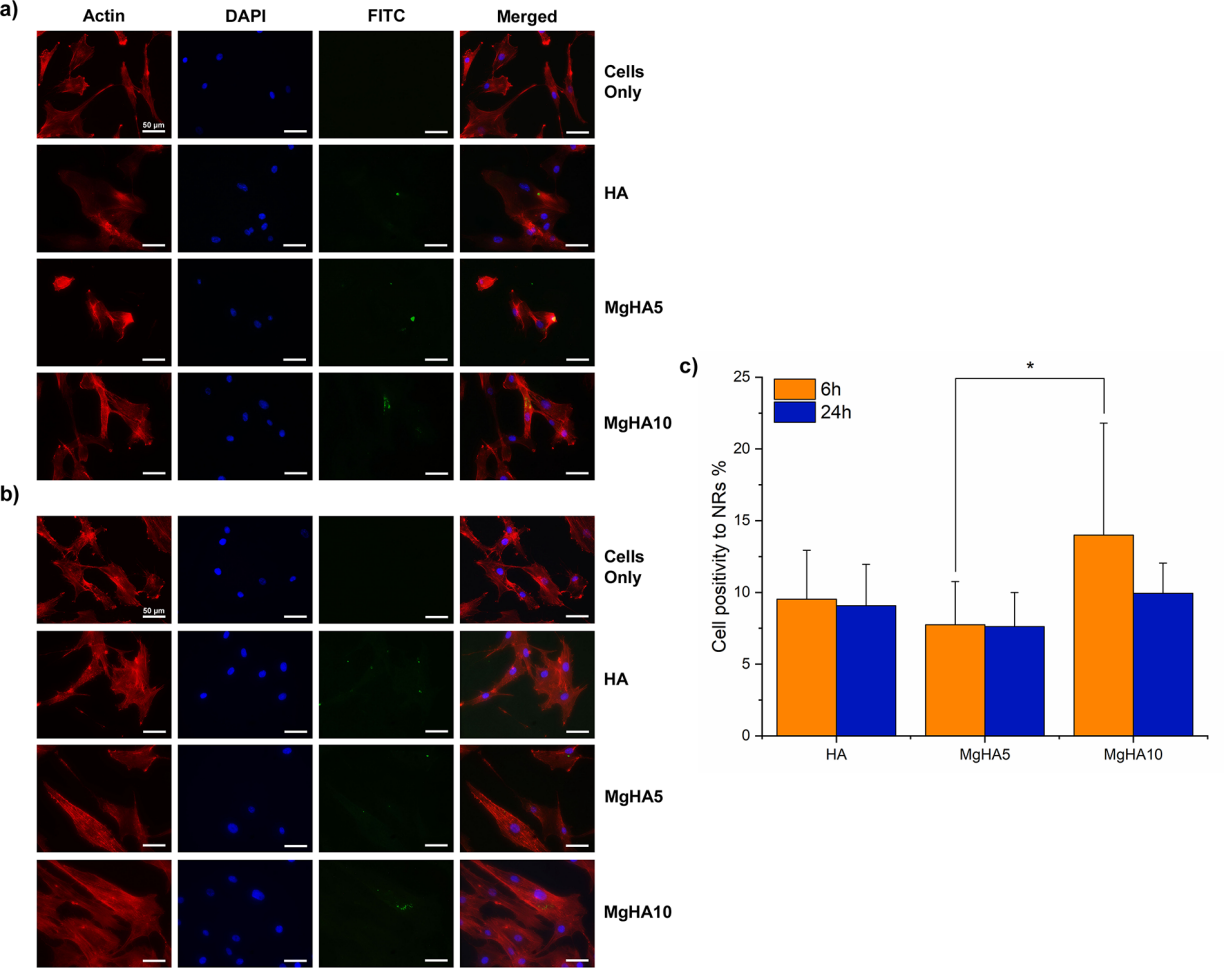
Visualization of the internalized FITC-conjugated NRs. No NRs were detected in cells only. All FITC-conjugated NRs were mainly detected surrounding the cell nucleus at 6 (a) and 24 h (b) of incubation, together with semiquantitative evaluation of cells positivity to FITC-NRs internalization (c). FITC-conjugated NRs in green, F-actin filaments in red, cell nuclei in blue. Scale bar 50 μm.

Among the samples tested, MgHA10 NRs showed the highest uptake, likely reflecting the alterations in the physical properties resulting from Mg^2+^ doping. Semiquantitative analysis of cells exhibiting fluorescence from FITC-labeled NRs further supported this finding (in Figure 7c). Smaller nanoparticles often internalize more efficiently because of reduced steric hindrance and easier membrane wrapping.^47^ In this study, Mg^2+^ incorporation results not only in smaller NRs but also in modifications of the surface chemistry, charge, and crystallinity. These combined effects alter protein corona formation and increase affinity for cell membranes, thus facilitating endocytosis.^18,48^ These combined properties make MgHA10 NRs particularly promising for functionalization and intracellular drug delivery. Further systematic studies, employing complementary labeling strategies and quantitative analyses, will be necessary to confirm these findings. Overall, this work offers an initial framework for correlating cytocompatibility with cellular internalization and identifying the most suitable NR candidates for subsequent DOX functionalization. However, further investigations will be necessary to more robustly and directly visualize the internalization of NRs and confirm their localization within the lysosomal compartment.

### 3.4 Osteogenic Gene Expression Profile

Due to its enhanced cytocompatibility and cell internalization, MgHA10 was chosen to further investigate its effect on the expression of five genes (RUNX-2, BMP-2, ALP, BGLAP, and SPP1) involved in the BM-MSCs’ osteogenic differentiation process,^25,26^ compared to HA and cells only. Figure 8 illustrates the gene expression profile of BM-MSCs following 7 and 14 days of incubation with HA and MgHA10 NR, supporting a clear induction of osteogenic differentiation, with an additional heatmap available (Figure S6) to visually illustrate the overall expression trends. At both time points, the presence of NRs did not have any statistically significant effect on the expression of the RUNX2 gene. RUNX-2 is activated within the first 7 days of osteogenic differentiation and functions as an upstream transcriptional regulator of BMP-2, as well as an activator of several downstream osteogenic genes, including ALP, BGLAP, and SPP1.^49,50^ Its expression typically increases during the early differentiation phase but tends to stabilize or slightly decrease as cells mature. Consistent with this observation, the stable expression of RUNX-2 over time in all the groups, together with a marked rise in BMP2 transcript levels starting from day 7, clearly indicated the activation of the BM-MSCs osteogenic commitment and differentiation process (Figure 8). Specifically, treatment with 50 μg/mL of MgHA10 NRs at days 7 and 14, and 200 μg/mL of HA at day 14, significantly enhanced BMP2 expression compared with cells only (p ≤ 0.01, p ≤ 0.001, and p ≤ 0.01, respectively). Notably, 50 μg/mL of MgHA10 NRs induced a greater BMP-2 upregulation than HA NRs at the same concentration after 14 days (p ≤ 0.01) (Figure 8). This trend aligns with the well-documented early and transient nature of BMP-2 expression—occurring concurrently with, or shortly after, RUNX-2 activation—and forming a positive feedback loop that reinforces RUNX-2 signaling.^51^

**Figure 8:**
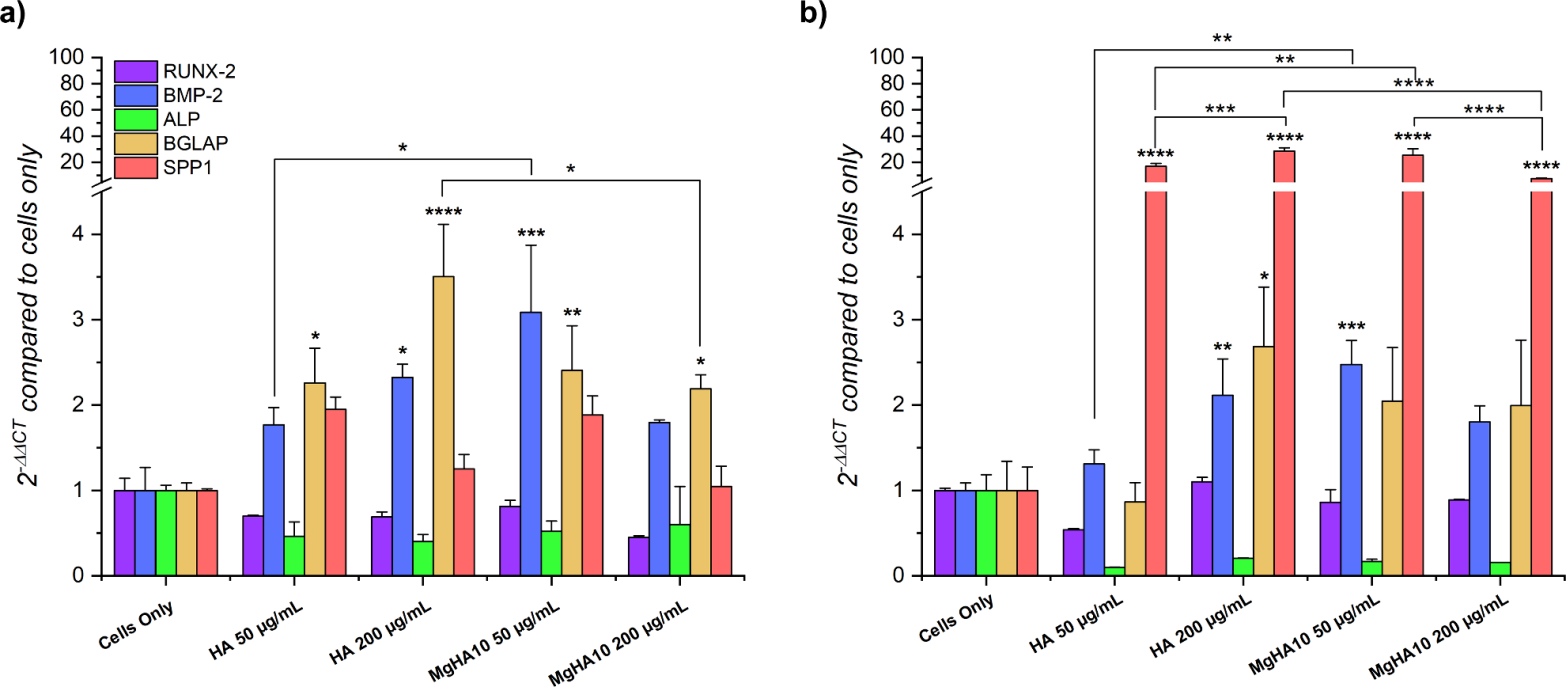
Gene expression of BM-MSCs after being cultured with HA and MgHA10 NRs. Relative quantification of RUNX-2, BMP-2, ALP BGLAP, and SPP1 genes with respect to cells only. The graphs show the fold change expression of the genes relative to cells only samples at day 7 (a) and 14 (b) of culture (mean ± standard error; *p value ≤ 0.05; **p value ≤ 0.01; ***p value ≤ 0.001; ****p value ≤ 0.0001). Asterisks placed above the histograms, without connecting lines, indicate statistical significance over the cells only sample.

To further assess the stage-specific commitments induced by the NRs, ALP and the late-stage osteogenic genes osteocalcin (BGLAP) and osteopontin (SPP1) were analyzed. ALP expression was downregulated compared to the cells only in all samples, and its expression further declined at day 14. Moreover, both BGLAP and SPP1 were upregulated at day 7, showing specific significant differences in the samples. Among the samples, 200 μg/mL of HA led to the highest BGLAP expression with respect to both control (p ≤ 0.0001) and MgHA NRs at the same concentration (p ≤ 0.05), closely followed by 50 μg/mL of MgHA (p ≤ 0.01 vs control). At day 14, BGLAP expression remained more or less stable, except for HA 50, while SPP1 expression drastically increased in all the NRs tested (p ≤ 0.0001). Once again, 200 μg/mL of HA again resulted in the strongest SPP1 upregulation, significantly higher than both control (p ≤ 0.0001) and MgHA NRs (p ≤ 0.0001). This was also here followed by 50 μg/mL of MgHA, which also demonstrated significantly elevated SPP1 levels compared with the control (p ≤ 0.0001) and HA NRs (p ≤ 0.05).

The expression profiles indicate that NRs induce the expression of late-stage osteogenic-related genes. In fact, Runx2 expression remained stable while ALP expression, which typically follows a Gaussian-like trend, rising during the premineralization phase and decreasing after matrix deposition,^52^ was downregulated, meaning that the expression peak probably occurred within the untested interval between days 7 and 14, or even earlier. This is consistent with the observed upregulation of BGLAP and SPP1, late markers of osteoblast maturation, which typically follow the ALP peak.^53,54^ Among the tested conditions, 50 μg/mL of MgHA10 elicited the strongest gene expression response, consistent with the enhanced ion release attributed to Mg^2+^ doping. Collectively, these findings support the role of Mg^2+^ ions incorporated into the apatite phase in promoting the progression of MSC toward late stages of osteogenic differentiation compared with undoped apatite.

### 3.5 Functionalization of HA and MgHA NRs with Doxorubicin (DOX)

To evaluate the capacity of the NRs to effectively deliver therapeutic drugs, MgHA10 NRs (hereinafter coded as “MgHA NRs”), were selected as the best in class to be loaded with doxorubicin (DOX), to be internalized in the human osteosarcoma cell line, and herein to release the drug intracellularly. The extent of DOX loading in percentage (calculated by applying eq 2) was higher in MgHA NRs (4.6 ± 0.2) compared to undoped HA (3.0 ± 0.3). Such phenomenon can likely be derived by the structural alterations induced by the inclusion of Mg^2+^ ions, which may have resulted in a partial disruption of the crystalline order in Mg-doped HA NRs surface.^19^ Consequently, the amorphization of the apatite surface could be considered responsible of the formation of charged surface sites linking DOX.^55^

The maintenance colloidal stability of NRs is crucial since it affects prolonged circulation in the bloodstream, internalization into targeted tumor, and drug release, which ultimately affect the effectiveness of DOX-functionalized nanocarriers in precision medicine. To assess such features with the obtained NRs, the hydrodynamic diameter of the synthesized NRs in pertinent fluids was measured (in Figure 9). Ultrapure water was used to evaluate intrinsic colloidal stability. A 67 mM citrate solution was employed at an optimized concentration to promote dispersion through its stabilizing effect on HA-based nanoparticles,^56,57^ and DMEM supplemented with 10% FBS was used to simulate biologically relevant conditions, including the presence of serum proteins. Both NRs exhibit excellent dispersibility in a 67 mM citrate solution, with the exception of MgHA NRs at day 0, likely due to a sudden change in the surface environment, causing transient agglomeration. As the system equilibrates with the citrate-containing medium, the dispersion progressively improves. Ultimately the data indicate that dispersing agents help preserve stable NR dispersion even at long-term time points, highly valuable for NRs dispersion storage. In ultrapure water, the dispersibility is maintained up to day 7, where sustained agglomeration occurs, particularly evident for MgHA NRs. To further assess NRs aggregation in cell culture media, DMEM was supplemented with 10% FBS, excluding pH indicators such as phenol red to prevent optical interference.^58^ Notably, at later time points, both NRs maintained a particle size below 200 nm in DMEM + 10% FBS, a key factor for enhanced cellular uptake and improved biodistribution.^59,60^ Interestingly, apparent anomalies in the particle size evolution were observed. For both samples, initial aggregation in FBS-supplemented DMEM was observed at day 1, likely due to the rapid adsorption of a soft protein corona, which transiently increased the apparent particle size. At later time points, partial rearrangement or desorption of loosely bound proteins may explain the subsequent size decrease, a behavior consistent with protein corona dynamics reported in the literature, though typically occurring at earlier time frames.^61,62^ Long-term monitoring revealed that these processes contribute to both transient aggregation and stabilization, providing insight into the evolving colloidal behavior of NRs in biologically relevant environments.

**Figure 9:**
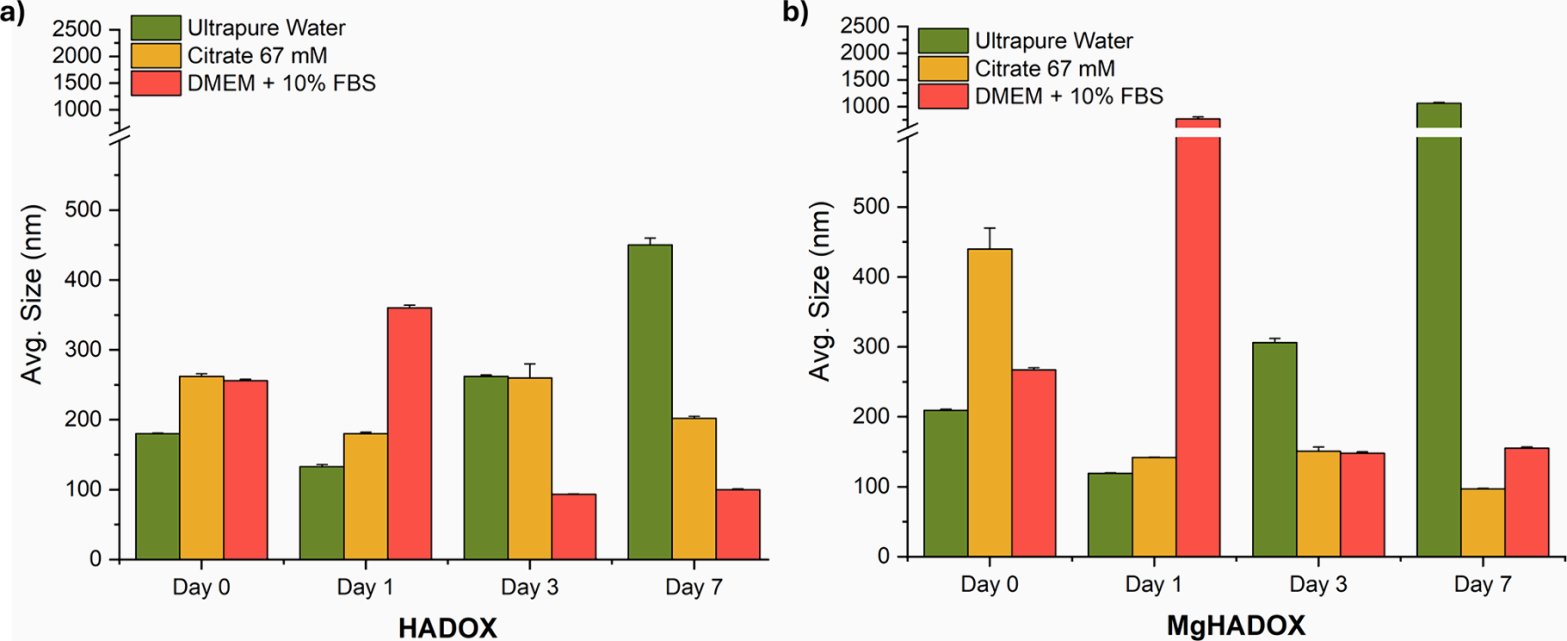
Colloidal stability of HA (a) and MgHA DOX-functionalized (b) NRs in various media.

### 3.6 DOX Release from NRs

DOX release from drug-loaded HA and MgHA NRs was calculated by applying eq 3. Low DOX release was observed at both pH values (Figure 10a), likely due to NRs aggregation in the release media, which limited NRs degradation. Nonetheless, this study clearly demonstrated substantial pH-dependent DOX release, with a significantly higher release at pH 5.0 compared to pH 7.4. After 7 days, a very low DOX extent was released at a physiological pH of 7.4. Conversely, under acidic conditions, drug release occurred more rapidly, attaining approximately 15% and 19% for HA and MgHA, respectively. Korsmeyer–Peppas model (KP) fitting further confirmed these trends (reported in Figure S7 and Table S2). In this model, the kinetic constant K_KP_ reflects the physicochemical and structural properties of the delivery system that govern drug release, including matrix degradability, drug diffusivity, and drug–matrix interactions. Higher K_KP_ values generally reflect a higher release rate.^63^ In the present work, higher K_KP_ was observed at pH 5.0 than at pH 7.4 for both NRs (as reported in Table S2), demonstrating pH-dependent release, and further, MgHADOX NRs exhibited higher K_KP_ than HADOX NRs under both conditions. The higher extent of drug release of MgHA NRs with respect to undoped HA NRs can likely be ascribed to the increased degradability of MgHA NRs, as confirmed by its ionic release (Figure 2). The drug release profile, quantified in μg/mL (as illustrated in Figure 10b), reveals a notable disparity in the DOX release kinetics between the two systems. The substitution of Ca^2+^ with Mg^2+^ in the apatitic structure leads to enhanced DOX adsorption onto the NR surface, leading to a higher initial drug loading. This increased loading capacity directly affects the subsequent release behavior, resulting in a sustained and potentially more controlled release profile. The synergistic effects of enhanced DOX loading and increased DOX release rates in MgHA NRs highlight the benefits of Mg^2+^ doping in improving drug delivery efficacy.

**Figure 10:**
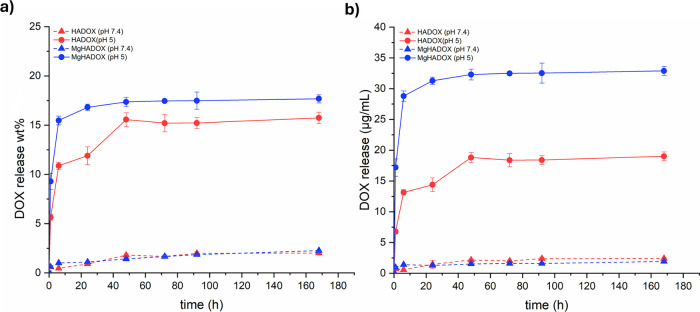
DOX releases from HA and MgHA NRs formulation expressed in wt % (a) and μg/mL (b).

From a structural standpoint, Mg^2+^ doping yields a dual function in altering the physicochemical properties of the HA NRs. Initially, this results in a reduction in the crystallinity of the apatitic core, a phenomenon that is well documented as an effect of the incorporation of Mg^2+^ ions into the HA lattice. The decrease in crystallinity is frequently ascribed to the different size of the ionic radius between Mg^2+^ and Ca^2+^, consequently disrupting the long-range order of the HA structure.^64^ Second, the Mg^2+^ ions are preferentially incorporated at the surface of the apatite structure, as an effect of the reduced lattice stability. These phenomena lead to surface amorphization, altering the physicochemical properties of NRs and potentially increasing the availability of active adsorption sites.^19,65,66^ As a consequence, an amorphous surface can enhance the adsorption of therapeutic agents, facilitating drug loading and improving its bioavailability. Moreover, lower crystallinity enhances NRs reabsorption, promoting their gradual removal from biological systems. This characteristic is essential for reducing long-term accumulation within cells after internalization, which may help minimize adverse cytotoxic effects. These findings highlight the suitability of Mg^2+^ doping as a method for optimizing the structural and functional characteristics of HA NRs as a drug delivery system.

The cytotoxic efficacy of free DOX and DOX-functionalized NRs at a concentration of 10 μg/mL (corresponding to 323 and 207 μg/mL of HA and MgHA NRs concentration, respectively) was evaluated on the human osteosarcoma cell line (Figure 11) and BM-MSCs (Figure S8) employing the MTT assay, in accordance with previous studies.^67,68^ The DOX-functionalized NRs showed significant cytotoxicity over time on healthy BM-MSCs as expected (reported in Figure S8). In the absence of any active targeting mechanism, the studied NRs display nonselective cytotoxicity, affecting both healthy and tumor cells alike.^69^ Under the tested conditions, nonfunctionalized HA and MgHA NRs did not yield any cytotoxicity on MG63 cells, reporting both a significantly higher cell viability compared to their relatives loaded with DOX at all time points (p value ≤ 0.0001). Moreover, no significant differences in cytotoxic activity were observed between free DOX and DOX-functionalized NRs, except at the early time points (days 1 and 2), when free DOX exhibited a slightly higher cytotoxic effect compared to HADOX, although the difference was modest (p ≤ 0.05). Such effects highlight the activity of both NRs to intracellularly deliver the DOX therapeutic agent. Up to day 2, no significant different cytotoxic effects were detected for the MgHADOX formulation with respect to free DOX. However, cellular uptake analysis revealed some differences in the intracellular localization of free DOX compared to DOX-functionalized NRs (Figure 12). Free DOX, after 6 h of incubation, is completely internalized and localized within the cell nucleus where it exerts its cytotoxic effect.^70,71^ Due to its amphiphilic nature, small size, and intrinsic fluorescence, DOX reaches the nucleus through passive diffusion across the plasma membrane. Once inside the cell, DOX preferentially accumulates in the nucleus by intercalating between DNA base pairs and stabilizing DNA–topoisomerase II cleavage complexes, thereby forming DNA–DOX adducts that effectively retain the drug within the nuclear compartment.^72^ Such adducts ultimately interfere with DNA replication and transcription, triggering cell cycle arrest and ultimately inducing apoptosis.^70^ Conversely, DOX-NRs show fluorescence signals both in the nuclei and as residual positivity in the cytoplasm. This suggests that although a lower concentration of DOX is released from NRs and reaches the nucleus it still manages to exert a cytotoxic effect comparable to that of free DOX. To better understand this behavior, fluorescence imaging showed that cells treated with DOX-NRs maintained a morphology similar to untreated controls, characterized by a well-spread cytoskeleton and normal nuclear appearance. This preservation of cytoskeletal integrity indicates that the formulation is initially biocompatible and further suggests a delayed onset of cytotoxic effects, likely due to the sustained release of DOX from the NRs. Further tests on different DOX concentration ranges (0.138–60 μg/mL) were carried out at 1 day of culture to investigate whether these differences in intracellular distribution may influence the anticancer efficacy at lower doses.

**Figure 11:**
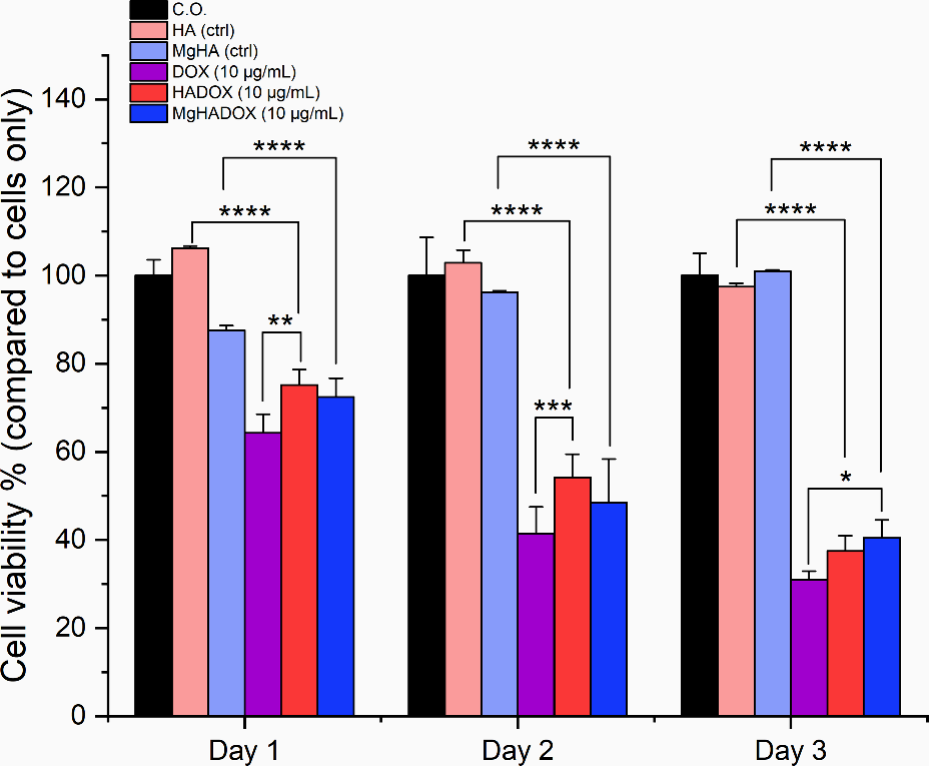
Cell viability analysis by MTT assay after 1, 2, and 3 days of MG-63 cell culture with NRs at fixed [DOX]:10 μg/mL and compared to free DOX at the same concentration as well as with equivalent amounts of unloaded NRs. Cells Only (C.O.) were used as control (mean ± standard error; *p value ≤ 0.05; **p value ≤ 0.01; ***p value ≤ 0.001; ****p value ≤ 0.0001). Asterisks placed above the histograms, without connecting lines, indicate statistical significance over cells only sample.

**Figure 12:**
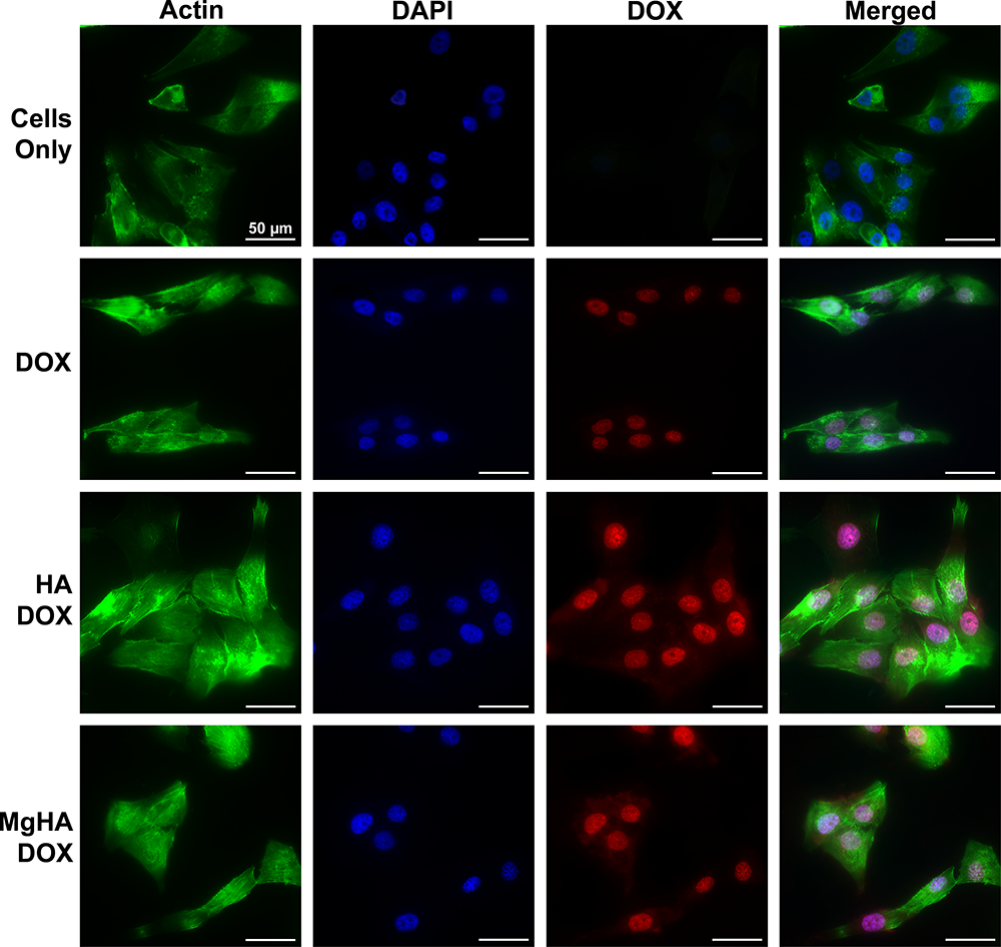
Intracellular localization of DOXO (in red color) in MG-63 cells after 6 h in the presence of free DOX, HADOX, and MgHADOX NRs with respect to Cells Only (CO). F-actin filaments in green, DAPI nuclei staining in blue. Bar = 50 μm.

The results, reported in Figure 13, demonstrated that at higher concentrations (3.75 to 60 μg/mL), free DOX generally displayed either higher or comparable cytotoxicity relative to the DOX-functionalized NRs, with MgHADOX showing significantly greater cytotoxic activity than HADOX at concentrations of 3.75 μg/mL (p ≤ 0.0001), 7.5 μg/mL (p ≤ 0.0001), and 15 μg/mL (p ≤ 0.01). As both unloaded HA and MgHA NRs showed no detectable cytotoxicity relative to the highest DOX concentration, it can be concluded that the NRs matrix is nontoxic and that the observed cytotoxicity arises from intracellular DOX delivery. At higher DOX concentrations, the cytotoxic response reaches a plateau and becomes largely independent of the delivery modality, which masks potential differences between NR-mediated and free DOX delivery.^70^ Conversely, at low concentrations (0.416 to 1.25 μg/mL), MgHADOX exhibited significantly higher cytotoxic activity compared to both free DOX (p ≤ 0.001) and HADOX (p ≤ 0.0001). In this low-concentration regime, NR-mediated delivery becomes particularly effective as it reduces the amount of DOX required to inhibit cell proliferation. The enhanced effect observed for MgHA NRs can therefore be attributed to their ability to promote DOX accumulation within the nucleus, unlike free DOX, which displays a more diffuse intracellular distribution. These findings highlight the advantageous properties of MgHADOX NRs, which demonstrate superior DOX loading capacity and faster degradation kinetics compared to undoped HA NRs, resulting in a more efficient and targeted release of the drug, even at low doses, but sufficient to induce strong cytotoxic effects. This work ultimately seeks to implement a biomimetic approach such as Mg^2+^ doping, which is extensively investigated in regenerative medicine, and is here applied as innovative DDS, as a promising and adaptable platform in the field of nanomedicine.

**Figure 13:**
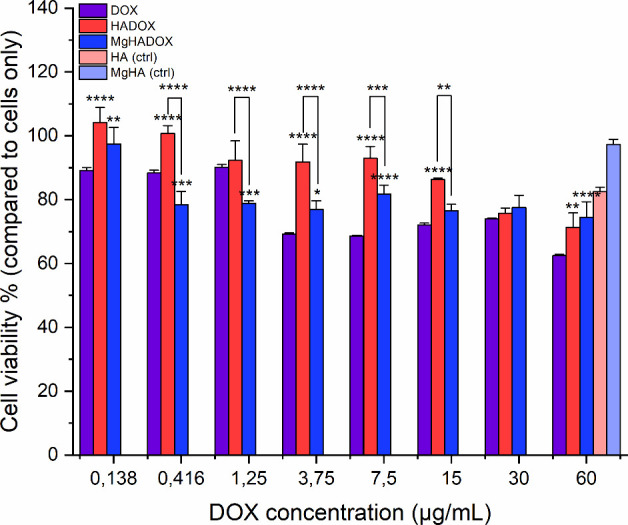
Cell viability analysis by MTT assay was after 1 day incubation of MG-63 cells with NRs at increasing DOX concentration and compared to free DOX at the same concentration and to unloaded HA and MgHA equivalents at highest DOX concentration. Values are reported by percentage (%) mean with respect to Cells Only (CO) values, used as control (mean ± standard error; *p value ≤ 0.05; **p value ≤ 0.01; ***p value ≤ 0.001; ****p value ≤ 0.0001). Asterisks placed above the histograms, without connecting lines, indicate statistical significance over free DOX sample.

## 4 Conclusions

The present study demonstrates the improved biocompatibility and efficiency of intracellular DOX delivery shown by Mg-doped apatite NRs as related to the low crystallinity, reduced particle size, and improved biodegradability, all effects induced by the Mg^2+^ doping. Increasing the extent of Mg^2+^ doping up to ∼6.5 mol % the long-term cell survival, drug loading capacity, and cell internalization are enhanced. Additionally, the NRs exhibit a pH-dependent DOX release profile, enabling more effective drug delivery in acidic environments—such as those found in endosomes. DOX-functionalized MgHA NRs demonstrated equivalent or even enhanced cytotoxicity against osteosarcoma cells in comparison with free DOX, especially at reduced doses, presumably due to the enhanced cellular absorption and intracellular release. In addition, we observed that the internalization of MgHA NRs enhances osteogenic gene expression; our results demonstrate that the use of MgHA NRs is a very promising approach for more effective cell-based therapies targeting dual anticancer/osteo-regenerative functions. This dual functionality is particularly relevant for designing integrated treatment techniques that leverage multifunctional NRs capable of potentially combining delivery of antitumoral agents and regenerative effects. The inherent cytocompatibility, the tunable properties elicited by Mg^2+^ doping, and the functional versatility of this system are the key points for designing integrated treatments in precision medicine.

## Supplementary Material



## Data Availability

Data will be made available on request.

## Notes

NRs crystallographic data, FTIR spectra, SEM dimensional analysis graphs, ionic release expressed in wt %, DOX adsorption optimization procedure, NRs proliferation assay with BM-MSCs line, heatmap of BM-MSCs expression of osteogenic-related genes with HA and MgHA NRs, DOX release profiles fitted with Korsmeyer–Peppas model, and DOX-NRs cell viability with BM-MSCs line (PDF)
