# Supplementary material for: Mg-Hydroxyapatite Nanorods for Dual Intracellular Doxorubicin Delivery and Osteogenic-Associated BM-MSC Responses
Source: ACS Appl Bio Mater. 2026 Feb 6;9(5):2602–17. doi: 10.1021/acsabm.5c02324 (PMC12958345; doi:10.1021/acsabm.5c02324)
Supplement: Supplementary file 1 [file mt5c02324_si_001.pdf]

# Supporting Information

# Mg-hydroxyapatite nanorods for dual intracellular doxorubicin delivery and osteogenic-associated BM-MSC responses

Federico Pupilli†, Giada Bassi†, Marta Tavoni†, Monica Montesi†, Anna Tampieri†, Simone Sprio†*

† Institute of Science, Technology and Sustainability for Ceramics – National Research Council of Italy (ISSMC-CNR), Faenza, Italy

* Corresponding author. E-mail address: simone.sprio@issmc.cnr.it

***Table S1.*** *Crystallographic data of the synthesized HA and MgHA NRs.*

| | a (Å) | c (Å) | c/a | Volume ($Å^3$) | $D_{av}$ | $D_{300}$ | $D_{002}$ | Shape factor | Splitting factor index (SFI) |
|---|---|---|---|---|---|---|---|---|---|
| **HA NRs** | 9.425 | 6.889 | 0.731 | 529.89 | $25.3 \pm 2.6$ | 30.6 | 67.4 | 2.20 | 8.15 |
| **MgHA5 NRs** | 9.419 | 6.876 | 0.730 | 528.27 | $21.6 \pm 2.2$ | 23.8 | 44.9 | 1.89 | 6.75 |
| **MgHA10 NRs** | 9.417 | 6.865 | 0.729 | 527.31 | $17.5 \pm 1.8$ | 21.9 | 35.1 | 1.60 | 6.21 |

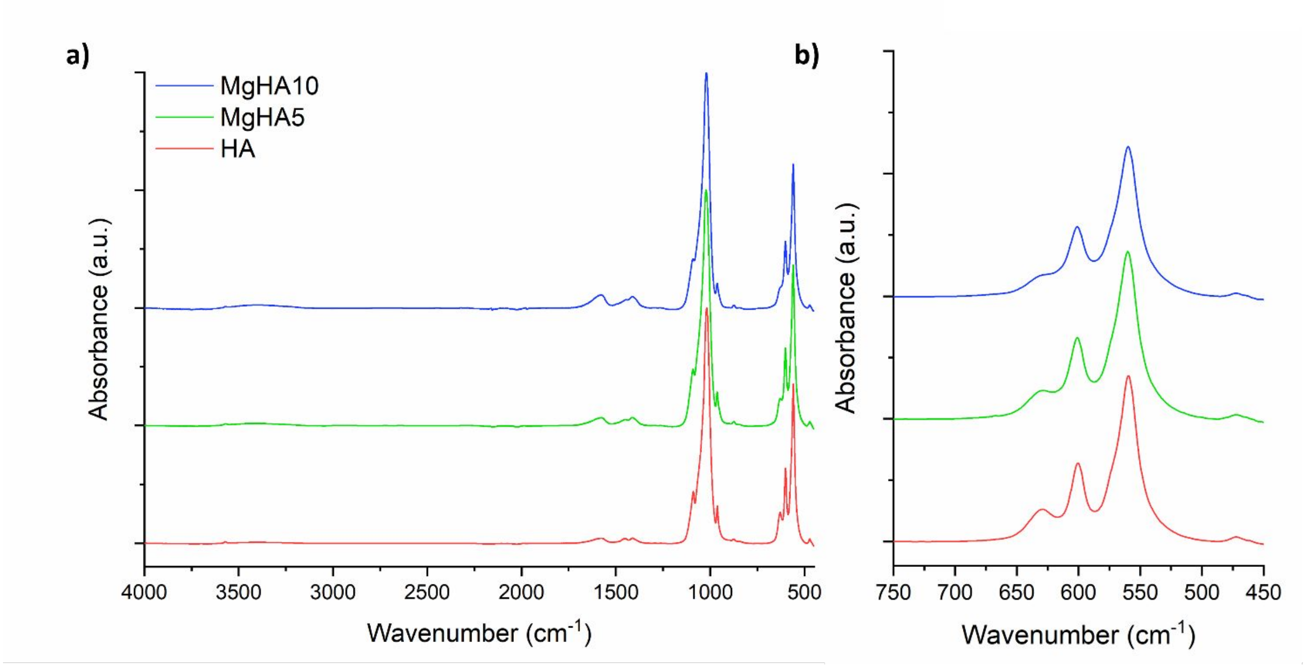


***Figure S1.*** *a) FTIR spectra of the as-synthesized NRs with b) zoom-in in the ν4(PO4) domain.*

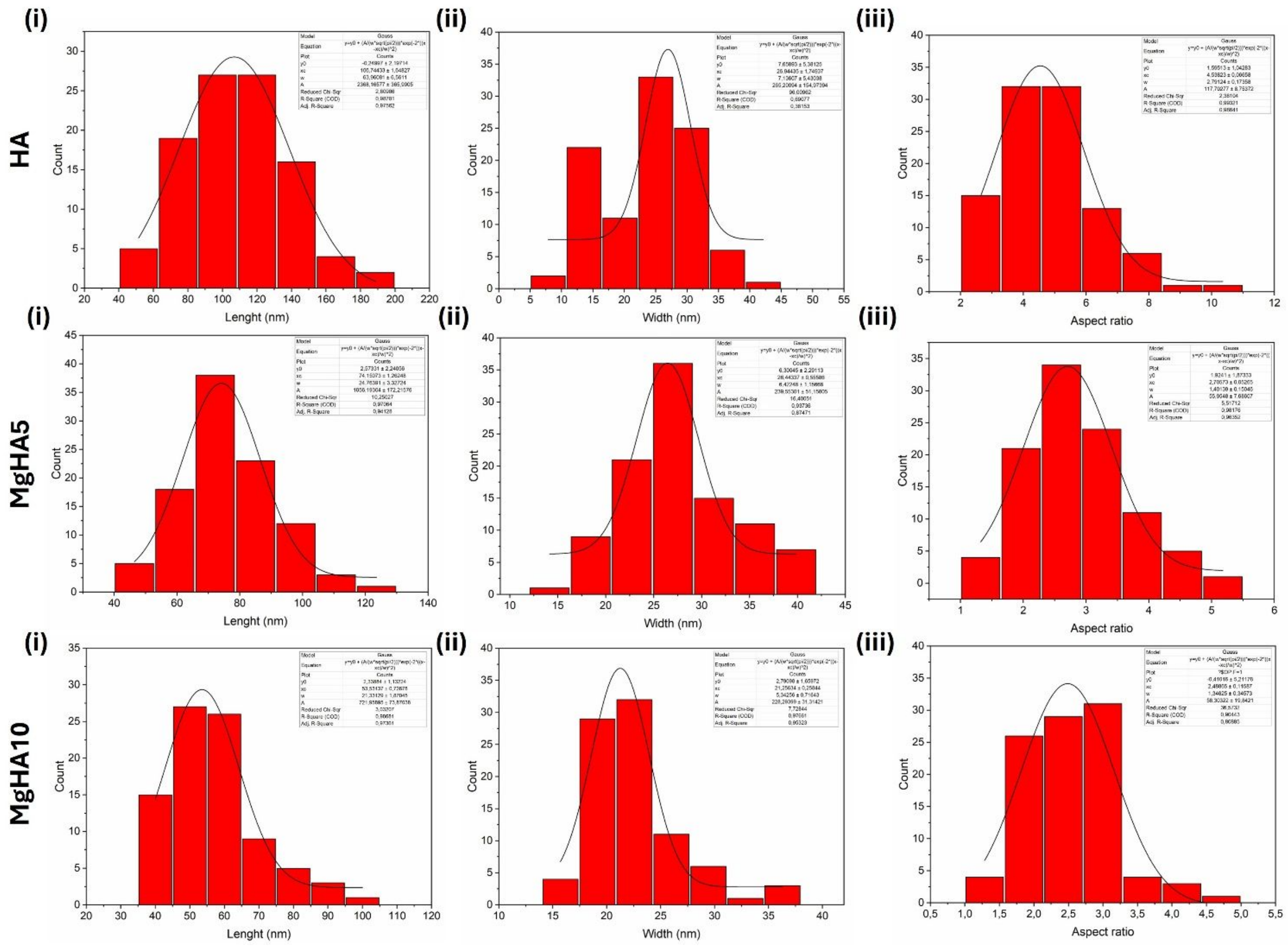


***Figure S2.*** *Dimensional analysis of length **(i)**, width **(ii)** and aspect ratio **(iii)** calculated from SEM micrographs of HA and $Mg^{2+}$-doped NPs.*

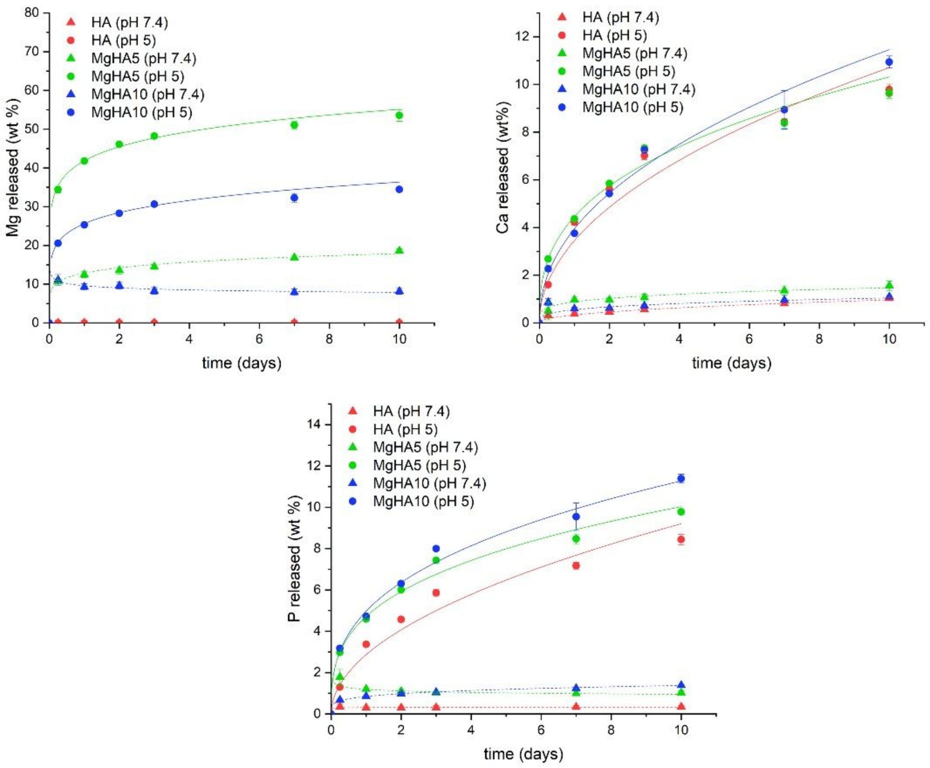


***Figure S3.*** *Ionic releases of the studied NRs at physiological (HEPES 10 mM buffer pH: 7.4; Acetate 10 mM buffer pH: 5).*

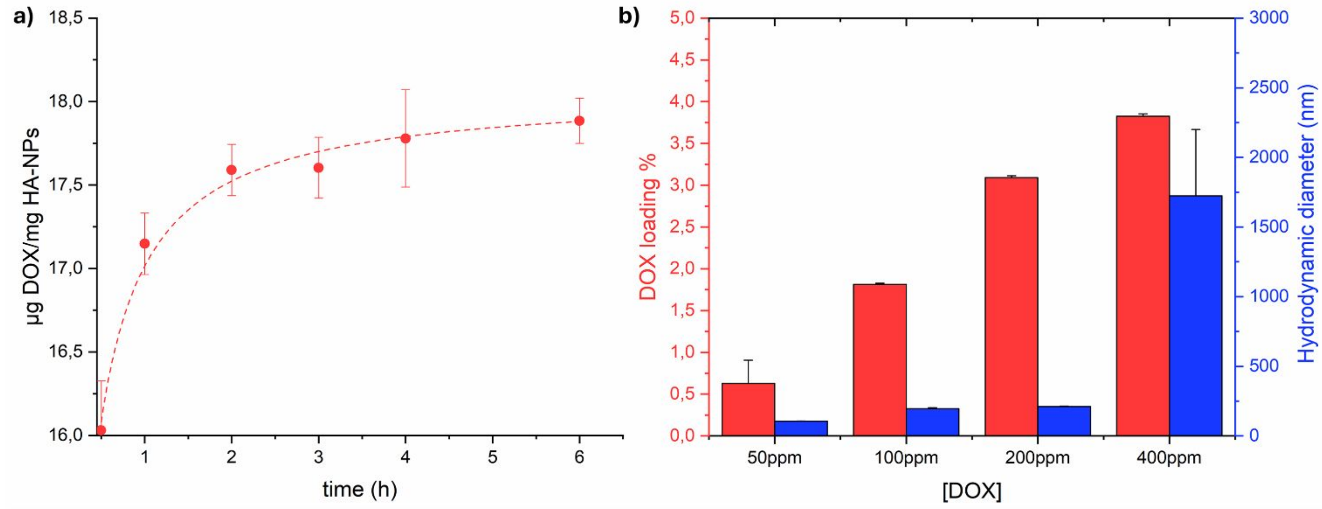


***Figure S4.*** *Optimization procedure for DOX adsorption. DOX adsorption kinetic* ***(a)*** *on undoped HA NPs, and loading evaluation at different nominal functionalization concentrations* ***(b)****.*

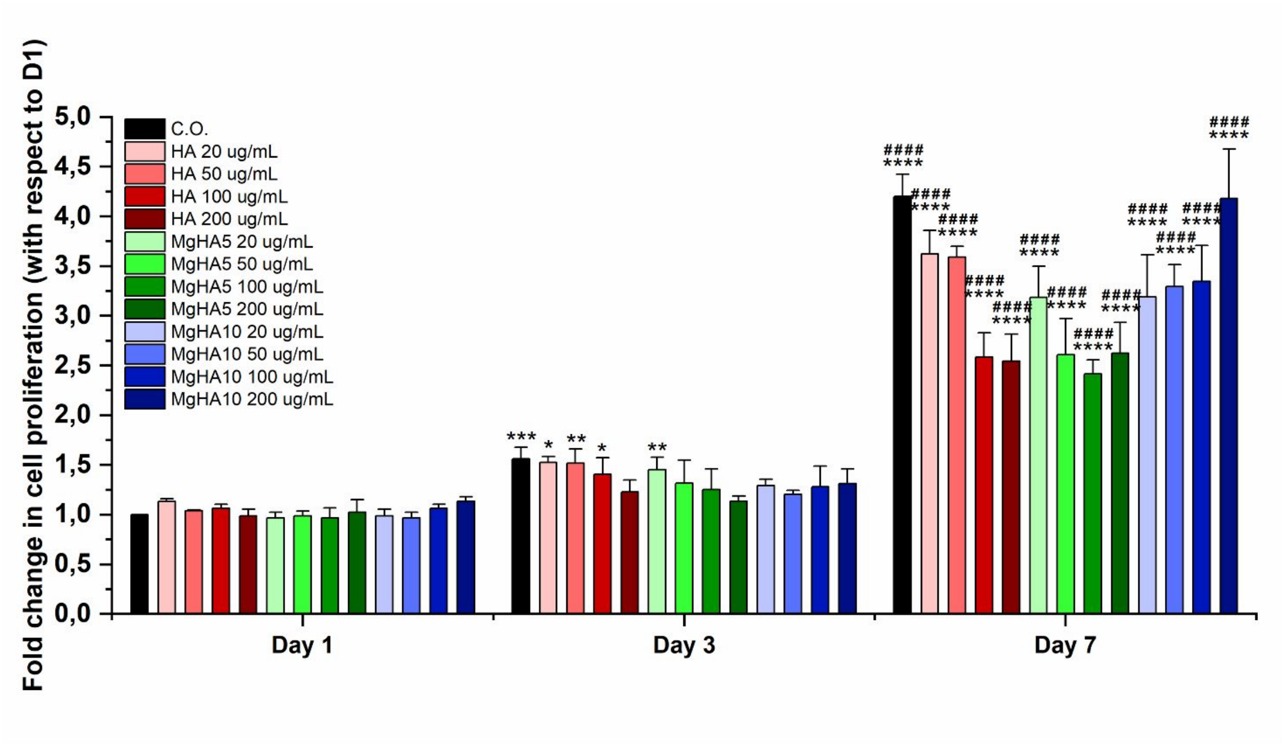


***Figure S5.*** *Cell proliferation analysis. MTT assay was performed after 1 day, 3 days and 7 days of BM-MSCs culture with 20 µg/ml, 50 µg/ml, 100 µg/ml and 200 µg/ml of HA (**a**), MgHA5 (**b**) and MgHA10 (**c**) NRs. Cells only (CO) served as the control, and fold change values were determined as normalization on day 1. (mean ± standard error; *p value ≤ 0.05; **p value ≤ 0.01; ***p value ≤ 0.001; ****p value ≤ 0.0001; * symbol denote significance relative to the fold change on day 1; # symbols denote significance compared with the fold change on day 3).*

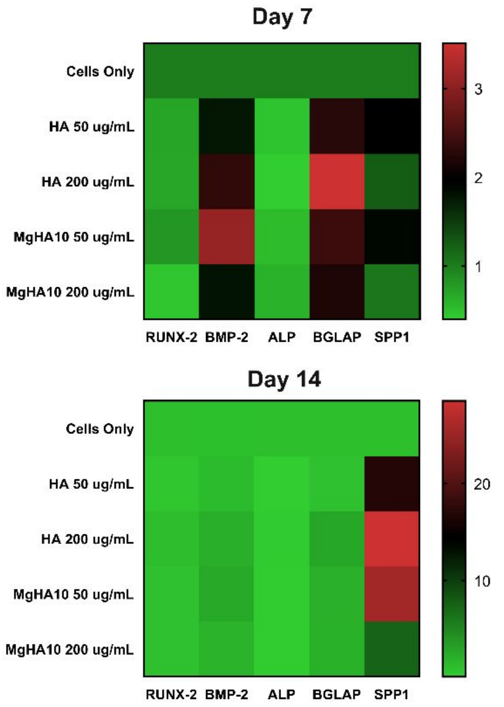


***Figure S6.*** *Heatmap representation of the relative expression levels of RUNX-2, BMP-2, ALP, BGLAP, and SPP1 genes with respect to cells-only controls. Gene expression values are shown as fold changes normalized to cells-only samples at day 7 (a) and day 14 (b) of culture. Color intensity reflects the magnitude of gene regulation (up- or down-regulation), with data expressed as mean values.*

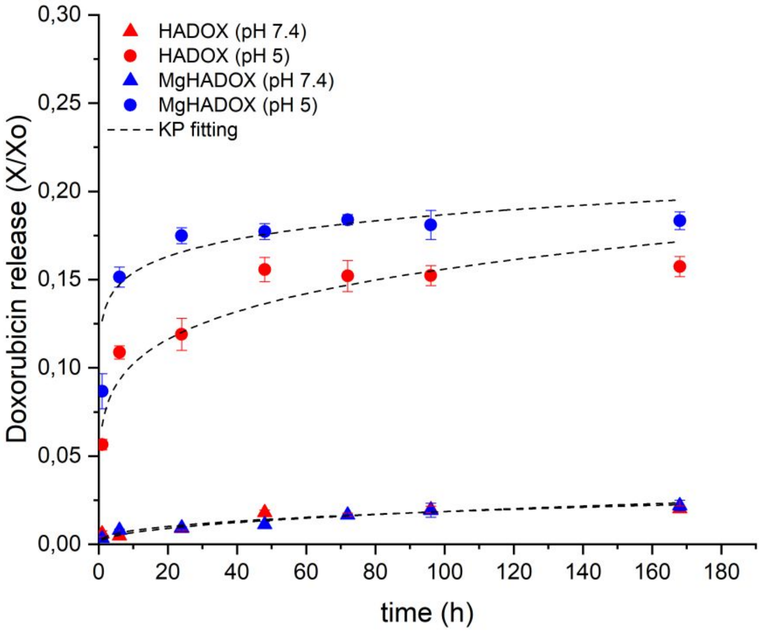


***Figure S7.*** *DOX release profiles from HA and MgHA NRs formulations, expressed as the fraction of DOX released relative to the initial DOX loading. The dotted black line represents the corresponding fitting with Korsmeyer–Peppas model.*

***Table S2.*** *Results of fitting DOX releases from HA and MgHA NRs with Korsmeyer-Peppas model.*

| | pH | $K_{(KP)}$ | n | $R^2$ |
|---|---|---|---|---|
| **HADOX NRs** | 7.4 | 0.0024 ± 0.0006 | 0.45 ± 0.07 | 0.914 |
| | 5 | 0.067 ± 0.007 | 0.18 ± 0.03 | 0.912 |
| **MgHADOX NRs** | 7.4 | 0.0033 ± 0.0004 | 0.37 ± 0.03 | 0.987 |
| | 5 | 0.13 ± 0.01 | 0.08 ± 0.02 | 0.746 |

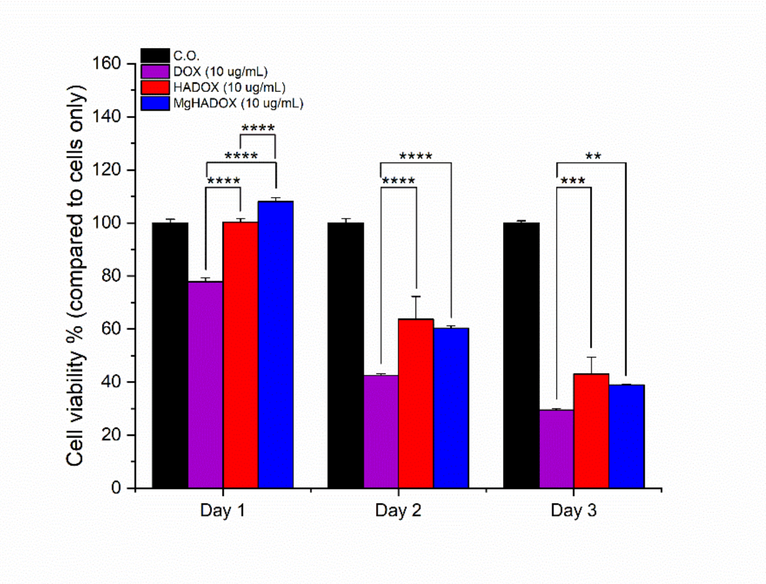


***Figure S8.*** *Cell viability analysis. MTT assay was performed after 1 day, 2 days and 3 days of BM-MSCs culture with NRs at fixed [DOX]:10 µg/mL and compared to free DOX at the same concentration. Cells Only (CO) were used as control (mean ± standard error; *p value ≤ 0.05; **p value ≤ 0.01; ***p value ≤ 0.001; ****p value ≤ 0.0001).*
